# Metabolomic Pathways Distinguishing Metabolically Healthy and Unhealthy Obesity from Normal-Weight: A Cross-Sectional Study

**DOI:** 10.3390/ijms27104555

**Published:** 2026-05-19

**Authors:** Neyla S. Al Akl, Olfa Khalifa, Abdelilah Arredouani

**Affiliations:** 1 Diabetes Research Center, Qatar Biomedical Research Institute (QBRI), Hamad Bin Khalifa University (HBKU), Qatar Foundation, Doha P.O. Box 34110, Qatar; nalakl@hbku.edu.qa (N.S.A.A.); okhalifa@hbku.edu.qa (O.K.); 2 College of Health and Life Sciences, Hamad Bin Khalifa University (HBKU), Qatar Foundation, Doha P.O. Box 34110, Qatar

**Keywords:** metabolically healthy obesity, metabolically unhealthy normal-weight, metabolic unhealthiness, metabolomics, adiposity

## Abstract

Metabolic health extends beyond BMI: some obese individuals remain metabolically resilient, while many normal-weight individuals exhibit hidden dysfunction. In Qatar, where obesity and metabolic disorders are widespread, understanding these divergent phenotypes is clinically paramount. This study maps the circulating metabolites and biological pathways associated with metabolic dysfunction. Clinical data from 6000 adults in the Qatar Biobank were used to determine obesity prevalence and classify metabolic health status among individuals with normal-weight and obese individuals. Comprehensive statistical analyses were conducted to determine associations between metabolic phenotypes and clinical parameters. Untargeted plasma metabolomics were performed in a subset of participants. Differential metabolite expression and pathway enrichment analyses were conducted to identify the distinct metabolic signatures underlying metabolic health. Mean age ranged from 30.9 ± 11.3 years in metabolically healthy normal-weight (MHNW) individuals to 46.0 ± 10.9 years in metabolically unhealthy obese (MUHO) individuals, with females predominating in the MHO group (65.2%). MUHO participants had higher rates of diabetes (21%), hypertension (34.7%), and insulin resistance (96.8%). Elevated C-peptide, uric acid, ferritin, and inflammatory markers were positively associated with metabolic unhealthiness, particularly in obese females, whereas estradiol and free thyroxine showed protective associations. Metabolomic profiling revealed distinct lipid and amino acid signatures differentiating MHO, MUHO, and MHNW phenotypes, with pathway analysis highlighting disruptions in lipid metabolism, amino acid pathways, and membrane transport. Metabolic heterogeneity in obesity reflects differences in coordinated biochemical regulation rather than adiposity alone. Identified metabolite signatures and ratios may serve as biomarkers of metabolic resilience and early risk stratification.

## 1. Introduction

Metabolic health is a crucial determinant of overall well-being, affecting both obese and non-obese individuals. It is now well established that obesity does not invariably correlate with poor metabolic health [1]. A subset of individuals with obesity exhibit a metabolically healthy phenotype referred to as metabolically healthy obese (MHO), characterized by preserved insulin sensitivity, favorable glucose regulation, and relatively normal lipid profiles [2]. Conversely, optimal metabolic status is not always observed in normal-weight individuals; some present with metabolic disturbances despite a normal BMI, a condition often termed “normal-weight obesity” and classified as metabolically unhealthy normal-weight (MUNW) [3].

This phenotypic variability underscores the complexity of metabolic health and the limitations of relying solely on BMI or body fat percentage to assess disease risk [1,4]. The MHO phenotype has emerged as a subject of considerable scientific interest due to accumulating evidence indicating its association with reduced incidences of cardiometabolic pathologies [4,5,6,7,8] and a lower risk of all-cause mortality relative to individuals classified as metabolically unhealthy obese (MUO) [9,10,11]. Furthermore, epidemiological and clinical studies have increasingly implicated the MHO phenotype in conferring a decreased susceptibility to neurodegenerative conditions, including Alzheimer’s disease [12,13], as well as to select malignancies, thereby challenging the traditional paradigm that equates obesity uniformly with heightened disease risk [14,15]. Nevertheless, the mechanisms that confer this protective metabolic profile remain elusive. Research to date has yielded inconsistent findings [16]. While individuals with MHO or MHNW phenotypes appear initially protected from metabolic complications, accumulating evidence suggests that this favorable status is often transient [17]. Many individuals eventually progress to metabolically unhealthy states, such as MUO or MUNW. This transition reflects the evolving nature of metabolic health, which is shaped by a complex interplay among chronic inflammation, genetic predisposition, gut microbiota diversity, adipose tissue distribution and expandability, and metabolic biomarkers [16,18]. Investigating the biological factors underlying metabolic health in obesity is crucial for improving disease prevention and management strategies.

In Qatar, the prevalence of overweight and obesity is among the highest globally, affecting approximately 78% of the adult population [19]. This excessive adiposity is strongly linked to a range of metabolic disorders, most notably type 2 diabetes (T2D) and prediabetes (PreD), which affect 15.6% and 17.7% of the population, respectively; both well above the global average [19,20]. These alarming rates reflect socio-economic transitions, including a shift toward energy-dense diets and increasingly sedentary lifestyles [21,22]. Given the magnitude of these interconnected conditions, understanding the pathophysiological mechanisms that differentiate metabolically healthy from unhealthy obese is especially important in these high-risk populations.

Metabolomics, the comprehensive analysis of small-molecule metabolites, offers a powerful approach to uncovering the molecular mechanisms that differentiate metabolically healthy from unhealthy phenotypes. It provides a dynamic snapshot of physiological states influenced by both genetics and environmental exposures [23]. This approach can reveal early biochemical signatures associated with metabolic health and disease risk, providing insights into the pathways that sustain metabolic health in obesity.

This study aimed to identify circulating metabolites associated with metabolic health across obese and normal-weight individuals, with particular emphasis on metabolically unhealthy phenotypes. We first quantified the prevalence of these phenotypes and examined their associations with some metabolic disorders. Subsequently, untargeted plasma metabolomics were employed to delineate distinct metabolic profiles underlying protective versus pathogenic states, providing mechanistic insight into metabolic health heterogeneity.

## 2. Results

### 2.1. Participants’ Characteristics and Cardiometabolic Profiles

Among the 6000 participants, the mean age was lowest in the MHNW and highest in the MUHO participants (30.9 ± 11 versus 46.0 ± 11; *p* < 0.001). Females were significantly predominant in the MHO (65.2%). Significant differences were observed in several cardiometabolic risk factors between MHO and MUHO individuals, as well as between MHNW and MUHNW subjects (Table 1). MHO participants had a more favorable cardiometabolic risk factor profile than MUHO participants, including lower fasting glucose (FPG), total cholesterol (TC), low-density lipoprotein cholesterol (LDL), triglycerides (TGs), and higher high-density lipoprotein cholesterol (HDL). Furthermore, liver enzymes alanine aminotransferase (ALT) and aspartate aminotransferase (AST) remained within normal ranges among all obese participants but were notably lower in the MHO group. HOMA-IR was elevated in both obese groups, but it was higher in the MUHO group. While BMI was modestly but significantly lower in MHNW compared to MUHNW participants, values in both groups remained within the normal range (BMI < 25 kg/m^2^), indicating the limited clinical relevance of this difference. Notably, MHNW individuals demonstrated more favorable metabolic profiles, including lower FPG, HbA1c, TC, LDL, and liver enzymes and systolic and diastolic blood pressure (SBP and DBP), and higher levels of HDL cholesterol. The prevalence of diabetes was significantly higher in the MUHO group (21%) compared to the MHO (13.2%), MUHNW (14.7%), and MHNW (2.2%) groups (*p* < 0.001). Similarly, the prevalence of PreD and IR was higher in the MUHO group (25.2% and 96.8%, respectively) and the MUHNW group (20.6% and 92.1%, respectively) compared to their metabolically healthy counterparts. The prevalence of hypertension showed a similar trend, with the MUHO group having the highest rate (34.7%), followed by the MUHNW group (23.5%). In contrast, the MHO and MHNW groups had significantly lower rates, at 22.9% and 4.7% respectively (*p* < 0.001). Additionally, hypertriglyceridemia was significantly more common in MUHO (84.1%) and MUHNW (80.9%) individuals, in sharp contrast to the more favorable lipid profiles observed in MHO and MHNW participants (*p* < 0.001). These findings highlight the distinct cardiometabolic risk profiles associated with metabolic health status, independent of obesity.

### 2.2. Differential Clinical Biomarkers Linked to Metabolic Unhealthiness in Obese and Normal-Weight Individuals

Logistic regression analysis identified the distinct clinical factors associated with the metabolically unhealthy phenotypes across obese and normal-weight individuals, with notable sex-specific differences. In obese females, inflammatory markers such as C-reactive protein and ferritin, along with metabolic indicators including C-peptide, potassium, and uric acid, were positively associated with metabolic unhealthiness (Table 2a). Protective associations were observed for estradiol, urea, and free thyroxine. Similarly, in obese males, elevated C-peptide, ferritin, alkaline phosphatase, uric acid, and albumin were linked to metabolic unhealthiness, whereas vitamin D, free thyroxine, bicarbonate, and estradiol appeared protective, as shown in Table 2b. Among normal-weight females, C-peptide showed a strong positive association with metabolic unhealthiness (Table 2c), alongside elevated TSH and uric acid levels. Estradiol and free triiodothyronine exhibited protective effects. In normal-weight males shown in Table 2d, elevated C-peptide and uric acid were positively associated with the metabolically unhealthy phenotype, while higher testosterone, free thyroxine, urea, and bilirubin were inversely associated, suggesting protective roles. Total protein was positively associated with metabolic unhealthiness in this group.

### 2.3. Differentially Expressed Metabolites and Their Clustering Patterns Between MHO and MUHO

**Figure 1:**
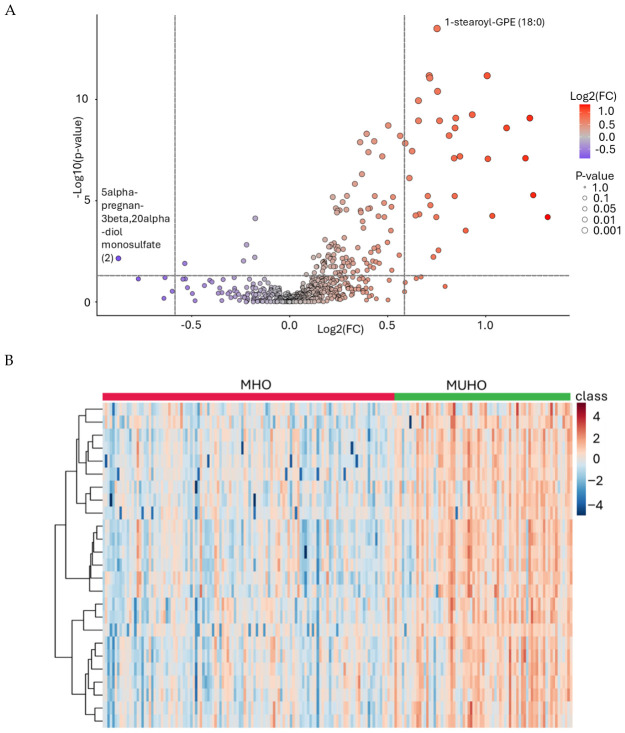
Differential plasma metabolite expression between MHO and MUHO groups. (**A**) The volcano plot illustrates the log_2_ fold change (x-axis) versus the −log_10_ adjusted *p*-value (y-axis) for all detected metabolites. Color represents a continuous gradient of log_2_(FC) (red: higher in MUHO; blue: lower in MUHO), while point size reflects the magnitude of statistical significance (*p*-value). The horizontal dashed line indicates the significance threshold (FDR = 0.05; −log_10_ ≈ 1.3), and vertical dashed lines indicate the fold change threshold (FC = 1.5; log_2_FC ≈ ±0.58). Metabolites located in the upper right and upper left regions exceed both thresholds, corresponding to significantly upregulated and downregulated metabolites in MUHO, respectively. (**B**) Heatmap representing supervised hierarchical clustering of the top 25 differentially expressed metabolites across MHO and MUHO individuals. Blue shading indicates lower expression, and orange/brown shading indicates higher expression in the MUHO group. * Indicates metabolites with putative identification based on high-confidence spectral matching but lacking confirmation by authentic standards.

Differentially expressed metabolites (DEMs) between MHO and MUHO were initially identified and visualized using volcano plots (Figure 1A), based on a fold change (FC) threshold of >1.5 and an adjusted *p*-value (FDR) of <0.05. Table 3 lists the top 20 metabolites showing significant differential expression, predominantly elevated in MUHO versus MHO samples. Notably, several glycerophosphoethanolamines (GPEs) and glycerophosphocholines (GPCs), such as 1-stearoyl-GPE (18:0) (FC = 1.68, FDR = 3.15 × 10^−14^) and 1-palmitoyl-2-linoleoyl-GPE (16:0/18:2) (FC = 2.01, FDR = 1.66 × 10^−11^), were significantly upregulated. Additionally, lipid metabolites like oleoyl-linoleoyl-glycerol (18:1/18:2) [2] showed a FC of 2.34 (FDR = 1.2 × 10^−9^). A few metabolites were downregulated in MUHO, such as 5alpha-pregnan-3beta,20alpha-diol monosulfate(2) (FC = −1.83; FDR = 0.007). These metabolite alterations highlight distinct changes in lipid metabolism associated with the metabolically unhealthy phenotype. Clustering analysis using the top 25 DEMs further highlighted distinct metabolic signatures between the groups. Notably, metabolites such as 1-palmitoyl-GPI (16:0), 1-linoleoylglycerol (18:2), 1-palmitoyl-2-linoleoyl-GPE (16:0/18:2), 1-linoleoyl-GPE (18:2)*, 1-palmitoyl-2-arachidonoyl-GPE (16:0/20:4)*, and 1-arachidonoyl-GPE (20:4n6)* were consistently elevated in the MUHO group, as shown in the heatmap (Figure 1B). These findings reinforce the differential regulation of key lipid metabolites associated with metabolic health status in obesity.

### 2.4. Chemometric and Enrichment Pathway Analysis Using MHO and MUHO Datasets

**Figure 2:**
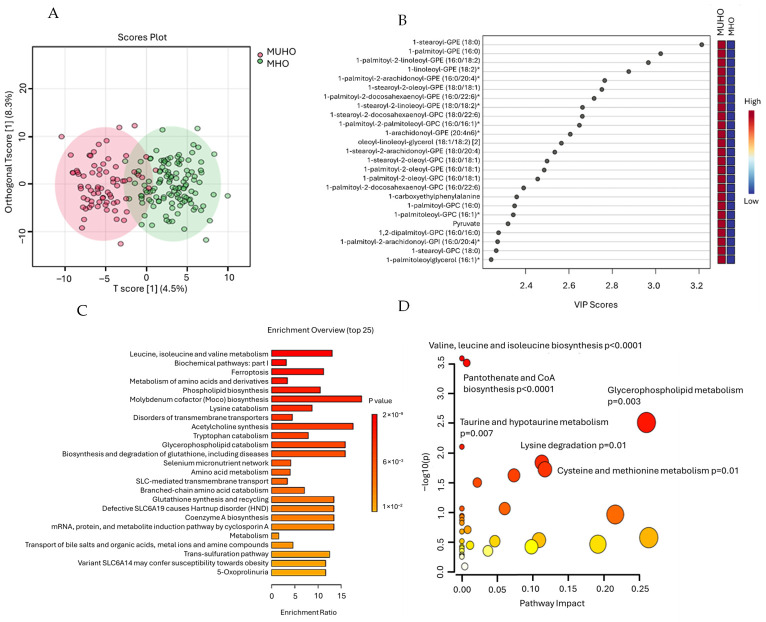
Chemometric analysis and pathway enrichment of metabolomic differences between MUHO and MHO groups. (**A**) Orthogonal Partial Least Squares Discriminant Analysis (OPLS-DA) score plot illustrating partial separation between MUHO and MHO samples. (**B**) Variable Importance in Projection (VIP) scores derived from the OPLS-DA model, highlighting the top 25 metabolites contributing to the separation between MHO and MUHO groups. (**C**) Bar plot representing the top enriched metabolic pathways identified using RaMP-DB, highlighting pathways significantly associated with differential metabolites (*p* < 0.05). (**D**) Pathway impact analysis based on KEGG and HMDB databases showing the relative influence of key metabolic pathways in distinguishing MUHO from MHO phenotypes. Larger circles indicate greater pathway impact, while the color gradient from white to red represents increasing statistical significance. Pathways with significant *p*-values (*p* < 0.05) are emphasized. * Indicates metabolites with putative identification based on high-confidence spectral matching but lacking confirmation by authentic standards.

To further investigate the metabolic distinctions between MUHO and MHO groups, we applied OPLS-DA to the respective metabolomic datasets. The OPLS-DA score plot (Figure 2A) shows a good separation between the two phenotypes, with only limited overlap. The model demonstrated robust explanatory and predictive capabilities, with cumulative R^2^Y and Q^2^ values of 0.736 (*p* < 0.001) and 0.36 (*p* < 0.0001), respectively (Supplemental Figure S1). These values indicate that the model explains 73.6% of the variance in the data and significantly predicts 36% of the variance better than would be expected by chance. A total of 139 key metabolites with VIP scores above 1, commonly used as the threshold [24], were identified as major contributors to group separation in Figure 2A and were selected for subsequent pathway enrichment analysis. The top 25 metabolites with the highest VIP are shown in Figure 2B, and the full list is shown in Supplementary Table S1.

Pathway enrichment analysis was conducted using the 139 metabolites and Receptor Activity-Modifying Protein Database (RaMP-DB), an integrative database that consolidates metabolite and lipid pathways from Kyoto Encyclopedia of Genes and Genomes (KEGG), Reactome, and WikiPathways. This comprehensive resource was selected to ensure broad, detailed coverage of metabolic and signaling pathways relevant to the underlying mechanisms that differentiate the cardiometabolic phenotypes investigated. A total of 22 metabolic pathways were significantly enriched based on raw *p*-values (<0.01) (Figure 2C). After adjusting for multiple comparisons using a false discovery rate (FDR) threshold of less than 0.1, three pathways remained statistically significant: Leucine, isoleucine, and valine metabolism (*p* < 0.001, FDR < 0.001), Biochemical pathways: part I (*p* < 0.001, FDR = 0.031), and Ferroptosis (*p* < 0.001, FDR = 0.076). Other significant pathways included Metabolism of amino acids and derivatives (*p* < 0.001), Phospholipid Biosynthesis (*p* = 0.003), Molybdenum cofactor biosynthesis (*p* = 0.004), Lysine catabolism (*p* = 0.005), Disorders of transmembrane transporters (*p* = 0.005), Acetylcholine synthesis (*p* = 0.005), and Tryptophan catabolism (*p* = 0.006). These pathways highlight key metabolic and signaling processes related to amino acid metabolism, lipid biosynthesis, and cellular transport, which may contribute to the observed phenotypic differences.

Complementing these results, pathway impact analysis using KEGG with HMDB-annotated VIP metabolites revealed significant perturbations (Figure 2D) in Valine, leucine, and isoleucine biosynthesis (*p* = 2.58 × 10^−4^, FDR = 0.012); Pantothenate and CoA biosynthesis (*p* = 3.06 × 10^−4^, FDR = 0.012). Glycerophospholipid metabolism also showed a significant raw *p*-value (*p* = 0.003) with an FDR just above the cutoff (FDR = 0.082). Other pathways with nominal significance included Taurine and hypotaurine metabolism (*p* = 0.008), Lysine degradation (*p* = 0.014), Cysteine and methionine metabolism (*p* = 0.019), Arginine and proline metabolism (*p* = 0.024), and Valine, leucine, and isoleucine degradation (*p* = 0.031).

### 2.5. Characterization of Differentially Expressed Metabolites and Their Clustering Patterns Between MHO and MHNW

**Figure 3:**
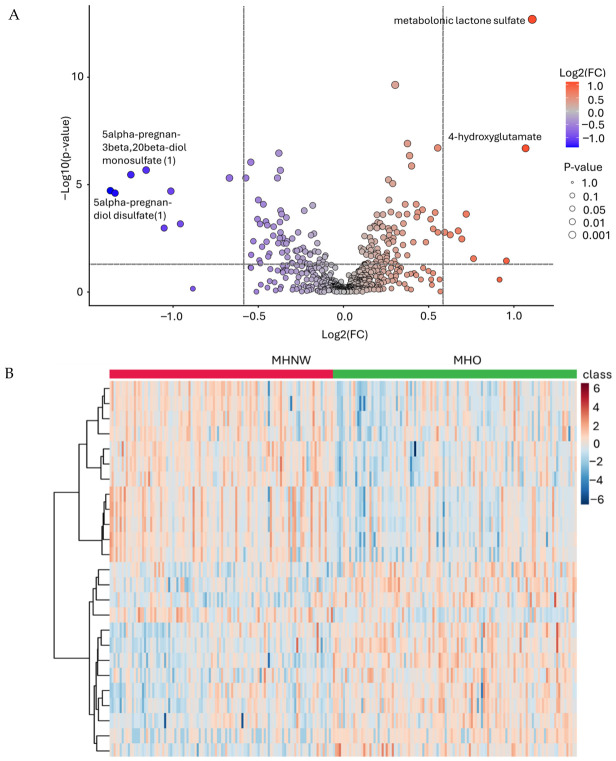
Differential plasma metabolite expression between MHO and MHNW groups. (**A**) The volcano plot illustrates the log_2_ fold change (*x*-axis) versus the −log_10_ adjusted *p*-value (*y*-axis) for all detected metabolites. Color represents a continuous gradient of log_2_(FC) (red: higher in MUHO; blue: lower in MUHO), while point size reflects the magnitude of statistical significance (*p*-value). The horizontal dashed line indicates the significance threshold (FDR = 0.05; −log_10_ ≈ 1.3), and vertical dashed lines indicate the fold change threshold (FC = 1.5; log_2_FC ≈ ±0.58). Metabolites located in the upper right and upper left regions exceed both thresholds, corresponding to significantly upregulated and downregulated metabolites in MUHO, respectively (**B**) The heatmap represents the supervised hierarchical clustering of the top 25 differentially expressed metabolites across MHO and MHNW individuals. Blue shading indicates lower expression, and orange/brown shading indicates higher expression in the MHO group.

DEMs between MHO and MHNW samples were initially identified and visualized using volcano plot analysis (Figure 3A). A total of 17 metabolites were found to be significantly altered, with 9 metabolites upregulated and 8 downregulated in MHO compared to MHNW (FC > 1.5, FDR < 0.05). Among the top upregulated metabolites were metabolonic lactone sulfate (FC = 2.15, FDR = 2.01 × 10^−13^), 4-hydroxyglutamate (FC = 2.09, FDR = 2.03 × 10^−7^), and 1-dihomo-linolenylglycerol (20:3) (FC = 1.64, FDR = 2.0 × 10^−4^). The downregulated metabolites included pregnanediol-3-glucuronide (FC = −2.23, FDR = 2.9 × 10^−6^), 5alpha-pregnan-3beta,20beta-diol monosulfate (1) (FC = −2.37, FDR = 3.44 × 10^−6^), and pregnolone sulfate (FC = −1.59, FDR = 5.0 × 10^−6^) (Table 4).

Hierarchical clustering of the DEMs further highlighted distinct metabolic profiles separating MHO from MHNW (Figure 3B). The heatmap demonstrated consistent downregulation of metabolites such as 1-(1-enyl-palmitoyl), 5alpha-pregnan-3beta, dehydroepiandrosterone sulfate, N-acetylglycine, pregnenediol sulfate, and androsterone sulfate in MHO individuals. In contrast, metabolites including 4-hydroxyglutamate, hydroxyasparagine, mannose, gamma-glutamylglutamine, and sphingomyelin were upregulated, highlighting a distinctive biochemical signature associated with metabolic health in obesity.

### 2.6. Chemometric and Metabolic Pathway Analysis Using MHO and MHNW Datasets

**Figure 4:**
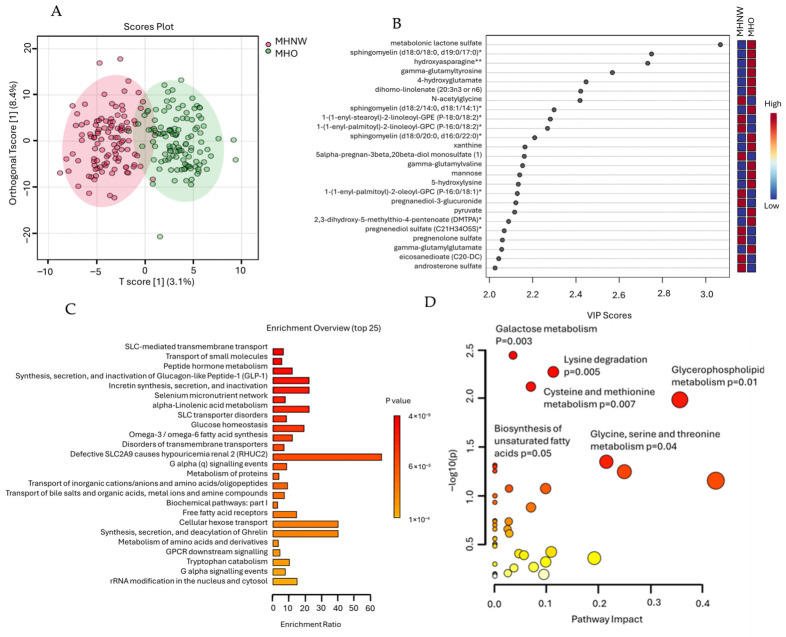
Chemometric analysis and pathway enrichment of metabolomic differences between MHO and MHNW groups. (**A**) Orthogonal Partial Least Squares Discriminant Analysis (OPLS-DA) score plot illustrating partial separation between MHO and MHNW samples. (**B**) Variable Importance in Projection (VIP) scores derived from OPLS-DA model, highlighting top metabolites contributing to separation between MHO and MHNW groups. (**C**) Bar plot representing top enriched metabolic pathways identified using RaMP-DB, highlighting pathways significantly associated with differential metabolites (*p* < 0.05). (**D**) Pathway impact analysis based on KEGG and HMDB databases showing relative influence of key metabolic pathways in distinguishing MHO from MHNW phenotypes. Larger circles indicate greater pathway impact, while the color gradient from white to red represents increasing statistical significance. Pathways with significant *p*-values (*p* < 0.05) are emphasized. * Indicates metabolites with putative identification based on high-confidence spectral matching but lacking confirmation by authentic standards. ** Indicates compounds for which authentic standards are unavailable; however, the identities are considered reasonably confident based on the available spectral and annotation data.

The OPLS-DA in Figure 4A shows a moderate separation between the MHO and MHNW groups, with slight overlap. Model robustness was confirmed by permutation testing (Supplemental Figure S2), yielding a Q^2^ value of 0.567 (*p* < 0.001, 1000 permutations) and an R^2^Y value of 0.774 (*p* < 0.001, 0/1000 permutations), indicating strong predictive ability and model validity. A total of 184 variables with a VIP score greater than 1 were identified and selected for further analysis of their associated pathways. The top 25 metabolites are shown in Figure 4B, and the full list is shown in Supplementary Table S2.

Pathway enrichment analysis using the 184 metabolites and the RaMP-DB revealed several significantly enriched pathways (Figure 4C). Key pathways exhibiting significant enrichment with both raw *p*-values and FDR below 0.1 included SLC-mediated transmembrane transport (*p* = 4.22 × 10^−9^, FDR = 6.2 × 10^−6^), transport of small molecules (*p* = 4.45 × 10^−9^, FDR = 6.2 × 10^−6^), peptide hormone metabolism (*p* = 5.61 × 10^−9^, FDR = 6.2 × 10^−6^), and the synthesis, secretion, and inactivation of Glucagon-like Peptide-1 (GLP-1) (*p* = 1.18 × 10^−8^, FDR = 7.81 × 10^−6^). Other notable enriched pathways included the selenium micronutrient network (*p* = 3.18 × 10^−8^, FDR = 1.76 × 10^−5^), alpha-linolenic acid metabolism (*p* = 1.42 × 10^−7^, FDR = 6.7 × 10^−5^), and SLC transporter disorders (*p* = 1.96 × 10^−7^, FDR = 8.1 × 10^−5^).

Additional pathways such as glucose homeostasis (*p* = 4.01 × 10^−7^, FDR = 1.48 × 10^−4^), omega-3/omega-6 fatty acid synthesis (*p* = 1.26 × 10^−6^, FDR =4.08 × 10^−4^), and disorders of transmembrane transporters (*p* = 1.35 × 10^−6^, FDR = 4.08 × 10^−4^) also showed significant enrichment. Further enriched pathways involved defective SLC2A9 linked to hypouricemia renal 2 (RHUC2), G alpha (q) signaling events, protein metabolism, and various transport mechanisms, all meeting the significance threshold (FDR < 0.05). Complementing these enrichment findings, pathway impact analysis identified several metabolic pathways with notable biological relevance (Figure 4D). Significant pathways included galactose metabolism (*p* = 0.0036), lysine degradation (*p* = 0.0053), and cysteine and methionine metabolism (*p* = 0.0075), reflecting key alterations in carbohydrate and amino acid metabolism. Additionally, glycerophospholipid metabolism (*p* = 0.0103) and glycine, serine, and threonine metabolism (*p* = 0.0447) showed meaningful pathway impacts, suggesting perturbations in lipid and one-carbon metabolism. These combined analyses highlight the multifaceted metabolic remodeling that distinguishes the MHO and MHNW groups.

### 2.7. Biomarker Analysis

The diagnostic utility of circulating metabolites was systematically evaluated using ROC curve analyses and area under the curve (AUC) metrics derived from multivariate logistic regression models. Models were internally validated via tenfold cross-validation and adjusted for potential confounders, including age and sex. Both individual metabolites and metabolite ratios were assessed for their discriminative performance.

**Figure 5:**
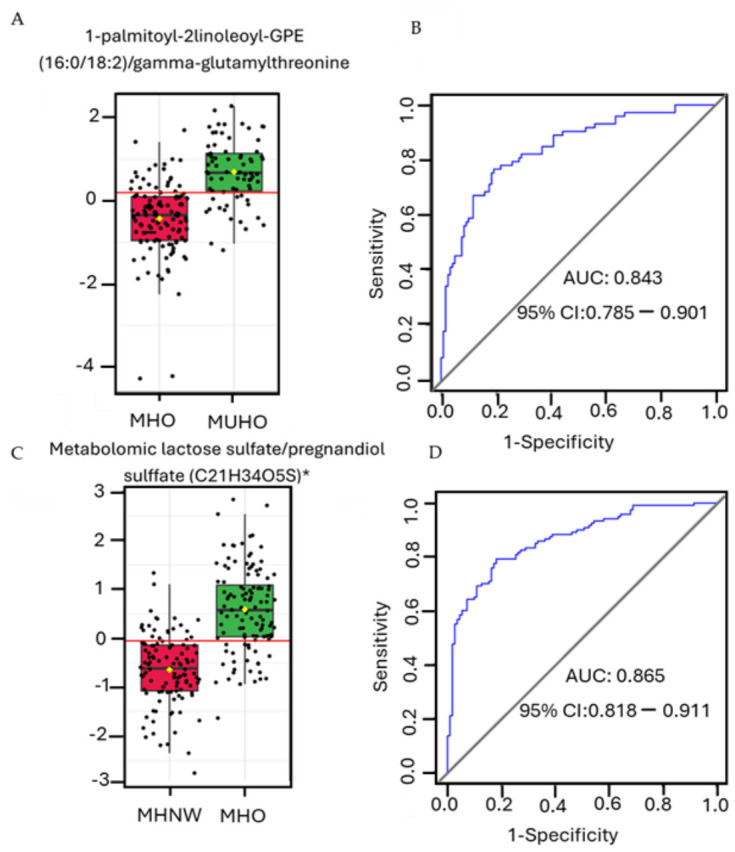
Biomarker Analysis of Metabolite Ratios Discriminating Metabolic Health Phenotypes. (**A**) The box plot illustrates the distribution of the metabolite ratio 1-palmitoyl-2-linoleoyl-GPE (16:0/18:2)/gamma-glutamylthreonine between MHO and MUHO individuals. (**B**) The receiver operating characteristic (ROC) curve demonstrates the discriminative performance of the logistic regression model based on this metabolite ratio in distinguishing MUHO from MHO, with area under the curve (AUC) indicated. (**C**) The box plot shows the distribution of the metabolite ratio of metabolonic lactone sulfate/pregnendiol sulfate (C21H34O5S)* between MHNW and MHO individuals. (**D**) The ROC curve depicts the performance of the logistic regression model using this ratio to discriminate MHO from MHNW, with the corresponding AUC displayed. * Indicates metabolites with putative identification based on high-confidence spectral matching but lacking confirmation by authentic standards.

ROC analyses revealed that metabolite ratios exhibited superior discriminatory power compared to single metabolites, achieving the highest AUCs for differentiating MUHO from MHO individuals, as well as MHO from MHNW individuals. Notably, the ratio of 1-palmitoyl-2-linoleoyl-GPE (16:0/18:2) to gamma-glutamylthreonine emerged as a robust predictor of MUHO versus MHO status (*p* < 0.001; odds ratio OR = 6.77), indicating a strong association between elevated values of this ratio and the metabolically unhealthy obese phenotype (Figure 5A). Logistic regression modeling demonstrated an AUC of 0.843 (95% CI: 0.785–0.901), with a sensitivity of 0.767 (95% CI: 0.767–0.864) and specificity of 0.808 (95% CI: 0.738–0.879), confirming the model’s robust discriminatory capacity (Figure 5B).

For the MHO versus MHNW comparison, the ratio of metabolonic lactone sulfate to pregnendiol sulfate (C21H34O5S) was identified as a significant biomarker (*p* < 0.001; OR = 8.39), strongly associated with the MHO phenotype (Figure 5C). The corresponding logistic model demonstrated excellent performance, with an AUC of 0.865 (95% CI: 0.818–0.911), a sensitivity of 0.792 (95% CI: 0.792–0.864), and a specificity of 0.818 (95% CI: 0.746–0.890) (Figure 5D).

Collectively, these findings highlight the distinct metabolite ratios that can effectively differentiate metabolic health phenotypes within and across obesity and normal-weight groups. The results suggest that perturbations in glycerophospholipid and steroid metabolism pathways may be potential mechanistic contributors to metabolic health heterogeneity.

## 3. Discussion

This study reveals critical metabolic heterogeneity within obese and normal-weight populations through the lens of untargeted plasma metabolomics. Our findings challenge the notion that adiposity alone defines metabolic health, revealing instead distinct biochemical signatures that delineate metabolic phenotypes. The pronounced divergence in cardiometabolic and biomarker profiles between metabolically healthy and unhealthy obese individuals, as well as between metabolically healthy obese individuals and normal-weight subjects, underscores the intricate biochemical networks that drive these contrasting metabolic states.

Consistent with prior literature, we observed that MUHO individuals had a significantly worse cardiometabolic profile compared to their MHO counterparts. Elevated FPG, lipid abnormalities, increased insulin resistance, and a higher prevalence of prediabetes, diabetes, and hypertension were evident in the MUHO group. These findings align with several studies demonstrating the adverse metabolic impact of obesity when coupled with insulin resistance and dyslipidemia [25,26,27]. Interestingly, despite comparable BMI levels, the MUHNW individuals also exhibited unfavorable cardiometabolic markers compared to their MHNW peers, underscoring that BMI alone does not fully capture metabolic risk [7].

The multivariable logistic regression analyses revealed distinct yet consistent biochemical correlates of metabolically unhealthy phenotypes across sex and obesity categories, with several markers showing robust, directionally similar associations. In both obese and non-obese groups, higher C-peptide and uric acid levels were strongly associated with increased odds of being metabolically unhealthy, reflecting compensatory hyperinsulinemia in response to peripheral insulin resistance, supporting a central role of altered purine metabolism in cardiometabolic risk [28,29,30]. Notably, the association between higher C-peptide and metabolically unhealthy status was particularly pronounced in normal-weight individuals, supporting the idea that early insulin resistance can precede overt adiposity and drive metabolic dysfunction independently of BMI. Conversely, hormonal regulators emerged as important protective factors; higher free thyroxine and estradiol concentrations were generally associated with reduced odds of metabolic unhealthiness. Thyroid hormones regulate mitochondrial oxidative metabolism, lipid oxidation, and glucose utilization [31], while estradiol enhances insulin sensitivity and protects against inflammation-induced metabolic dysfunction [32], all supporting the critical role of endocrine regulation in maintaining metabolic homeostasis [33,34,35]. Inflammatory and iron-related markers, including C-reactive protein, ferritin, and total iron-binding capacity in females, and ferritin and alkaline phosphatase in males, showed positive associations with metabolically unhealthy obesity, highlighting the contribution of low-grade inflammation and disturbed iron turnover to adverse metabolic profiles [36,37]. Elevated ferritin reflects increased systemic iron stores and has been strongly linked to oxidative stress, mitochondrial dysfunction, and insulin resistance [37]. Additional sex-specific patterns were observed, including positive associations between TSH and total protein, and an inverse association between bilirubin and metabolically unhealthy non-obese status, indicating that subtle differences in thyroid axis activity, protein metabolism, and antioxidant capacity may modulate metabolic risk beyond adiposity alone.

Untargeted metabolomic profiling revealed profound alterations in lipid metabolism between metabolic phenotypes. Differential expression analysis between MHO and MUHO individuals revealed a striking enrichment of dysregulated lipid species, predominantly lysophospholipids and related glycerophospholipids. The volcano plot and hierarchical clustering demonstrate that these metabolites segregate individuals into two clearly distinct metabolic clusters, supporting the notion that obesity-related metabolic health is strongly determined by qualitative differences in circulating lipids rather than adiposity alone. This observation aligns with recent reports indicating that dysregulated glycerophospholipid metabolism is a hallmark of insulin resistance and T2D [38,39,40]. Remarkably, multiple upregulated metabolites identified in MUHO contained polyunsaturated fatty acids such as linoleic acid (18:2) and arachidonic acid (20:4), including 1-palmitoyl-2-arachidonoyl-GPE and 1-stearoyl-2-arachidonoyl-GPE. These lipid subsets are biologically significant because arachidonic acid is a precursor of proinflammatory eicosanoids, including prostaglandins and leukotrienes [41], which contribute to chronic low-grade inflammation [42], a defining feature of metabolically unhealthy obesity [43]. Elevated phospholipids enriched in arachidonic acid may therefore reflect increased inflammatory lipid signaling and membrane phospholipid turnover in MUHO individuals. This proinflammatory environment contributes to chronic inflammation, which interferes with insulin signaling, thereby impairing glucose uptake and metabolic dysfunction [44]. Of note, the steroid metabolite 5alpha-pregnan-3beta,20alpha-diol monosulfate was significantly reduced in MUHO individuals. Reduced levels of steroid sulfates may indicate impaired steroid metabolism and altered endocrine regulation in metabolically unhealthy individuals. Steroid sulfates also serve as reservoirs for active steroid hormones, and their dysregulation may contribute to altered metabolic and inflammatory responses associated with insulin resistance [45]. Consistently higher levels of multiple lysophosphatidylethanolamine (GPE), lysophosphatidylcholine (GPC), and lysophosphatidylinositol (GPI) species in the MUHO group, alongside the alterations in the described glycerolipids and the steroid sulfate, point to perturbations in membrane remodeling, phospholipase activity, and lipid signaling pathways that are closely linked to insulin resistance, low-grade inflammation, and heightened cardiometabolic risk.

Increasing evidence suggests that metabolically unhealthy obesity is associated not only with systemic metabolic dysregulation but also with early vascular alterations. In this context, non-alcoholic fatty liver disease (NAFLD), which is highly prevalent in individuals with MUHO [46], has been linked to increased carotid intima-media thickness (IMT), a marker of subclinical atherosclerosis [47]. This association is thought to be mediated by shared mechanisms including insulin resistance and chronic low-grade inflammation, as demonstrated in studies showing that both NAFLD severity and inflammatory mediators are associated with increased IMT in obese individuals [47,48]. The lipid alterations identified in the present study, particularly the enrichment of arachidonic acid-containing glycerophospholipids and lysophospholipids, may contribute to endothelial dysfunction through enhanced inflammatory signaling and oxidative stress [49,50]. These findings provide a mechanistic framework linking metabolic dysregulation to vascular remodeling and suggest that the vascular alterations observed in MUHO are not solely attributable to excess adiposity but rather reflect the impact of underlying metabolic and inflammatory disturbances.

Enrichment Pathway Analysis using metabolites from oPLSDA results indicates that the separation between MHO and MUHO is driven predominantly by disturbances in amino acid and lipid metabolism, with a central role for branched-chain amino acids (BCAAs). Elevated or reprogrammed leucine, isoleucine, and valine pathways, together with broader amino acid metabolism and multiple branched-chain catabolic and disorder pathways, point to impaired BCAA handling that is associated with insulin resistance, mitochondrial stress, and cardiometabolic risk in obesity in the literature, highlighting their role as both biomarkers and mediators of metabolic dysfunction [51,52,53]. Perturbations in phospholipid and glycerophospholipid biosynthesis/catabolism, bile acid and organic anion transport, and SLC-mediated transmembrane transport pathways further implicate altered membrane remodeling, lipoprotein trafficking, and nutrient/solute transport as contributors to the adverse metabolic phenotypes. Notably, ferroptosis also emerged as a significantly enriched pathway that distinguishes MUHO from MHO, linking lipid dysregulation, iron metabolism, and oxidative stress. Ferroptosis has been implicated in the pathogenesis of obesity, insulin resistance, and T2D through mechanisms involving oxidative stress and lipid peroxidation [54]. Iron availability is a key determinant of ferroptotic susceptibility, as excess iron promotes reactive oxygen species generation and lipid damage [55]. These findings align with our logistic regression results, which show associations between iron-binding markers and metabolically unhealthy obesity, supporting a role for iron-dependent oxidative mechanisms in the metabolic deterioration observed in MUHO.

When comparing MHO to MHNW individuals, unique metabolite differences emerged. Among the most strongly upregulated metabolites in MHO was metabolonic lactone sulfate. According to Das et al. [56], metabolonic lactone sulfate was identified as a novel metabolite associated with cardiometabolic health and metabolic dysfunction, with higher levels linked to increased adiposity, insulin resistance, dyslipidemia, and elevated blood pressure. Its elevation suggests that even in the absence of overt metabolic disease, individuals with metabolically healthy obesity exhibit underlying metabolic adaptations or early biochemical changes associated with increased adiposity. This supports the concept that MHO represents an intermediate metabolic state characterized by compensatory metabolic remodeling rather than complete metabolic normality [56]. In parallel, the observed increase in 4-hydroxyglutamate and gamma-glutamyl amino acid derivatives further supports alterations in redox regulation and glutathione metabolism [57,58]. Elevated glutathione-related metabolites in MHO suggest increased antioxidant activity, possibly reflecting a compensatory response to higher the oxidative stress associated with increased adiposity compared to MHNW. This aligns with evidence that mitochondrial and oxidative stress signaling pathways are activated early in obesity, even before clinical metabolic abnormalities emerge [59]. Lipid metabolites provided additional insight into this adaptive state. Elevated monoacylglycerols such as 1-arachidonylglycerol and 1-palmitoleoylglycerol indicate increased lipid turnover and membrane remodeling, reflecting the dynamic handling of excess lipid substrates in obesity. These lipid intermediates are closely linked to metabolic signaling pathways and insulin action, highlighting their role in maintaining metabolic flexibility under increased energetic load [60]. Notably, 1-arachidonoylglycerol (1-AG) is an isomer of 2-arachidonoylglycerol (2-AG), a major endogenous endocannabinoid that regulates metabolic and inflammatory signaling through cannabinoid receptors [61]. While 2-AG represents the biologically active signaling form, 1-AG is a more stable, less active isomer that arises from acyl migration or the degradation of 2-AG, demonstrating endocannabinoid turnover [62]. Elevated 1-AG likely indicates increased flux through endocannabinoid pathways, because the endocannabinoid system plays a central role in lipid metabolism and energy homeostasis activation [63]. Higher circulating arachidonoylglycerols in MHO can reflect adaptive lipid remodeling and metabolic flexibility. However, continuous alterations in endocannabinoid-related lipid metabolism has been linked to obesity-associated insulin resistance, as circulating 2-AG levels are elevated in insulin-resistant obese individuals and correlate with metabolic dysfunction [64]. These changes suggest early metabolic adaptations preceding dysfunction. Furthermore, elevated levels of the modified amino acid hydroxyasparagine reflect increased protein turnover, mitochondrial metabolic activity, and oxidative stress, which are key features of early metabolic impairment, even in metabolically preserved obesity. Metabolomic studies have demonstrated that perturbations in amino acid metabolism, including asparagine-related pathways, are associated with impaired glucose homeostasis and increased cardiometabolic risk long before the onset of detectable diabetes [58,65]. In contrast, the consistent reduction in several steroid sulfate metabolites, including pregnenolone sulfate and multiple pregnanediol derivatives, suggests altered steroid hormone metabolism and endocrine adaptation in response to increased adiposity. Pregnenolone sulfate plays an important role in both metabolic and cognitive regulation [66]. In obesity, altered pregnenolone sulfate levels reflect changes in steroidogenesis and endocrine adaptation, which influence insulin sensitivity, inflammation, and metabolic homeostasis [67]. In the central nervous system, it has been linked to cognitive impairment in obesogenic environments [68]. Together, these findings indicate that pregnenolone sulfate links endocrine metabolic regulation with cognitive function, reflecting systemic adaptation to metabolic stress. Collectively, these findings demonstrate that MHO is characterized by coordinated metabolic adaptations involving enhanced antioxidant defenses, mitochondrial metabolic activity, lipid remodeling, and endocrine regulation, reflecting a metabolically adaptive state that maintains metabolic stability despite increased adiposity while potentially representing an intermediate stage preceding metabolic deterioration not totally equivalent to normal-weight individuals.

Chemometric analysis further demonstrated that MHO represents a metabolically distinct state compared to MHNW individuals. The OPLS-DA score plot revealed a clear separation between the groups, indicating that even in the absence of detectable metabolic disease, obesity is associated with tangible alterations in systemic metabolism. The enrichment of pathways related to solute carrier (SLC)-mediated transport pathways highlights the altered cellular uptake and distribution of metabolites, reflecting increased metabolic flux and nutrient handling in obesity. These transporters regulate the intracellular availability of amino acids, glucose, and lipids, and their modulation plays a critical role in maintaining metabolic balance under conditions of increased substrate availability [69,70]. Similarly, enrichment of glucagon-like peptide-1 (GLP-1) synthesis and signaling pathways suggests that adaptive endocrine responses preserve insulin secretion and glucose regulation [71,72], thereby maintaining metabolic health in MHO individuals, potentially reflecting compensatory mechanisms or early metabolic remodeling [73,74]. Lipid pathways were also significantly enriched, including alpha-linolenic acid metabolism, omega-3 and omega-6 fatty acid synthesis, and glycerophospholipid metabolism. These pathways are essential for membrane composition, lipid signaling, and inflammatory regulation [75,76]. Such changes may reflect adaptive lipid-handling mechanisms that enable individuals with MHO to accommodate increased lipid storage while maintaining metabolic stability. Notably, the involvement of selenium micronutrient networks suggests a multifaceted interplay between nutritional and biochemical factors in maintaining metabolic health in obesity [77]. In fact, the enrichment of selenium-related pathways highlights the critical role of selenium-dependent antioxidant enzymes, including glutathione peroxidases, in regulating oxidative stress and maintaining metabolic homeostasis. This supports the concept that enhanced antioxidant defense mechanisms contribute to metabolic resilience in MHO [78,79]. Additionally, amino acid metabolism, including lysine degradation, glycine, serine, and threonine metabolism, and cysteine and methionine metabolism were significantly enriched in MHO. These pathways are closely linked to mitochondrial function, antioxidant defense, and one-carbon metabolism [80,81,82]. Cysteine and glycine are essential precursors for glutathione synthesis, the primary intracellular antioxidant, and the increased metabolism of these amino acids suggests enhanced glutathione production and the activation of antioxidant defense mechanisms to maintain cellular redox balance [83]. These findings are consistent with the elevated gamma-glutamyl metabolites observed in our DEMs and further support the presence of adaptive antioxidant responses in MHO. Collectively, metabolically healthy obesity is characterized by coordinated metabolic adaptations involving lipid remodeling, enhanced antioxidant defenses, altered amino acid metabolism, and adaptive endocrine and transport mechanisms. These metabolic adjustments likely represent compensatory responses that help preserve metabolic stability in the face of increased lipid storage and metabolic demand. However, these changes demonstrate that MHO is not metabolically identical to normal-weight health but rather reflects a metabolically adaptive state that may influence long-term metabolic risk and susceptibility to progression towards metabolically unhealthy obesity.

Given the limited sample size of MUHNW individuals with available metabolomics data (n = 9), pathway enrichment analysis comparing this group to MHO obese participants should be interpreted with caution and presented as exploratory (Supplemental Material Figures S3 and S4). Despite these limitations, the analysis suggested potential differences in key biochemical pathways, including glucose homeostasis, amino acid metabolism, and membrane transport processes. Of particular interest were alterations in glycine, serine, and threonine metabolism, as well as in trans-sulfuration and one-carbon metabolic pathways, which have been previously implicated in redox balance and methylation reactions critical to metabolic regulation [84,85]. While these preliminary findings may suggest that metabolic unhealthiness in normal-weight individuals could be driven by disruptions in amino acid related pathways independent of adiposity, they require validation in larger cohorts. These observations are consistent with emerging evidence that metabolic dysfunction can occur in the absence of obesity and underscore the importance of investigating metabolic health beyond BMI categories.

Our biomarker analysis identified metabolite ratios with strong discriminative potential for distinguishing metabolic health phenotypes. The ratio of 1-palmitoyl-2-linoleoyl-GPE to gamma-glutamylthreonine was significantly associated with the MUHO phenotype, supporting the relevance of glycerophospholipid metabolism in metabolic dysregulation [58]. In contrast, the ratio of metabolonic lactone sulfate to pregnendiol sulfate was strongly linked to the MHO phenotype compared to MHNW individuals, suggesting a distinctive metabolic adaptation. These findings are consistent with previous studies that underscore the role of steroid metabolites and lactone derivatives in modulating insulin sensitivity, inflammation, and overall metabolic homeostasis [56,86]. In line with these observations, logistic regression models incorporating these metabolite ratios demonstrated robust discriminatory performance, with AUC values derived using 10-fold cross-validation. While internal cross-validation reduces overfitting, validation in independent cohorts will be important to confirm generalizability prior to any potential clinical application.

Limitations of our study include the cross-sectional design, which restricts causal inference, and the small sample size of the metabolomics subset, which may affect generalizability. Nevertheless, the integration of detailed clinical phenotyping with advanced metabolomic analysis in a large population-based cohort provides strong evidence of the biochemical distinctions underpinning metabolic phenotypes.

In conclusion, this study demonstrates that metabolic health in obesity is defined by adaptive biochemical regulation rather than adiposity alone. Metabolically healthy obesity represents a state of metabolic resilience characterized by coordinated adjustments in lipid remodeling, redox balance, mitochondrial function, and endocrine regulation that helps preserve systemic homeostasis despite excess adiposity. However, the elevation of metabolites such as metabolonic lactone sulfate, previously linked to cardiometabolic risk, suggests that MHO is not metabolically equivalent to normal-weight but rather reflects an intermediate state marked by early metabolic adaptation and increased metabolic demand. The identification of metabolite ratios integrating lipid, amino acid, and steroid metabolism, specifically in the ratios of 1-palmitoyl-2-linoleoyl-GPE to gamma-glutamylthreonine and metabolonic lactone sulfate to pregnanediol sulfate, provides novel composite biomarkers that capture functional metabolic status more effectively than individual metabolites. These ratios reflect the integrated balance between membrane lipid turnover, antioxidant capacity, intermediary energy metabolism, and steroid hormone regulation, offering valuable insight into the interconnected metabolic networks underlying metabolic resilience and dysfunction. Collectively, these findings highlight that metabolic heterogeneity in obesity is driven by differences in metabolic flexibility and adaptive capacity rather than body weight alone. Future longitudinal and mechanistic studies are needed to determine whether these metabolomic signatures predict metabolic deterioration or sustained metabolic stability and to evaluate their potential for improving early risk stratification and enabling precision prevention strategies in metabolic disease.

## 4. Methods

### 4.1. Study Participants and Assessment of Phenotypes

**Figure 6:**
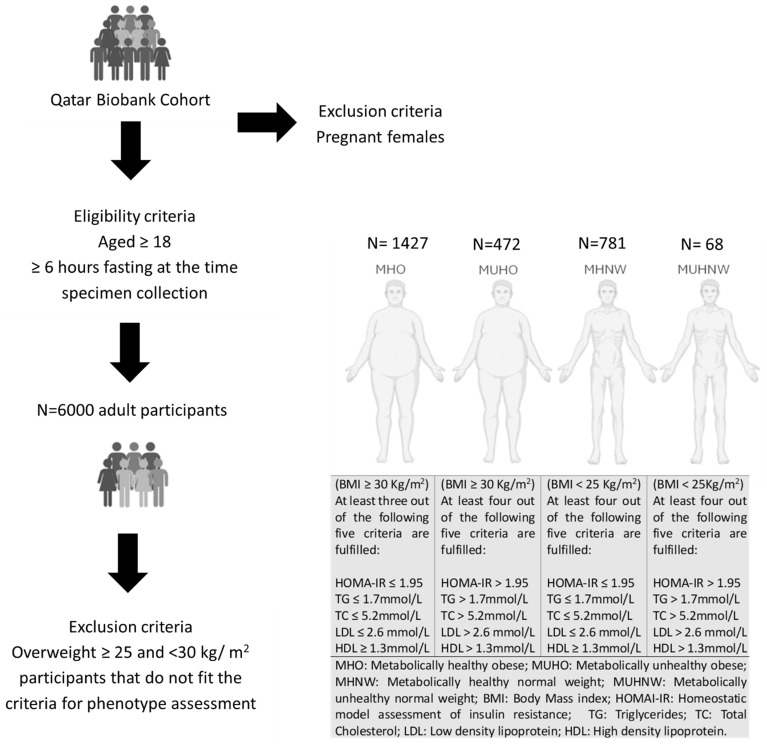
**Study Design Flow Chart.** Schematic illustrating participant recruitment and categorization into four sub-phenotype groups based on metabolic health and weight.

For this cross-sectional study, clinical, demographic, and anthropometric data from 6000 participants in the Qatar Biobank (QBB) cohort were obtained. The study’s inclusion criteria included individuals aged 18 years or older who had fasted for at least 6 h prior to specimen collection. Pregnant females were excluded from this study. Participation was contingent upon individuals providing informed consent, thereby permitting the collection and use of their data for research purposes, as managed by the QBB. Metabolic health determinants were used to estimate the prevalence of diabetes, PreD, IR, and hypertension in the general population. Diabetes and PreD were defined according to the American Diabetes Association (ADA) criteria. Participants with a fasting plasma glucose (FPG) ≥ 7.0 mmol/L (126 mg/dL) were classified as having diabetes, while those with FPG between 5.6 and 6.9 mmol/L (100–126 mg/dL) were considered to have PreD [87,88]. IR was assessed using the Homeostasis Model Assessment of Insulin Resistance (HOMA-IR) index calculated as follows: HOMA-IR = (fasting insulin [µU/L] × fasting glucose [mmol/L])/22.5 [89]. A threshold of ≥1.95 was applied to define IR, based on prior literature [90] including Karelis et al. [91] where similar values were used to define insulin sensitivity in metabolic phenotyping. This threshold is consistent with our previous studies [92], and was applied in the absence of established population-based cut-off values for HOMA-IR in Qatar or other GCC countries. Based on the phenotype assessment, participants were further categorized into MHO, MUHO, MHNW, and MUHNW to facilitate the analysis. Initial classification was based on BMI; obese having a BMI ≥ 30 kg/m^2^ or normal-weight having a BMI between 18.5 and 24.9 kg/m^2^ [91]. Further stratification was based on the metabolic criteria outlined in the National Cholesterol Education Program’s Adult Treatment Panel III report (ATP III) for lipid profiles [93], as summarized in Figure 6.

### 4.2. Metabolomic Profiling

Metabolomics data were acquired for 312 participants, stratified into the following subgroups: MHNW (110), MHO (120), MUHNW (9), and MUHO (73). These participants were part of a larger cohort of 3000 individuals randomly selected by QBB for metabolomic profiling. Data were generated using the Metabolon platform (https://www.metabolon.com), which detects and quantifies hundreds of known metabolites across multiple biochemical classes, including amino acids, lipids, carbohydrates, vitamins, peptides, nucleotides, acylcarnitines, glycerophospholipids, sphingomyelins, and xenobiotics. A total of 1160 metabolites were initially identified. As we are only interested in endogenous metabolites, Xenobiotics were excluded from the analysis. Missing metabolite values accounted for less than 4.5% of the data matrix and were imputed using the lowest detected value, consistent with the assumption that missingness reflected low-abundance metabolites below the detection limit. Data were then log-transformed and scaled prior to statistical analysis. After data cleaning and filtering, a total of 567 endogenous metabolites remained, distributed across the following classes: amino acids (159), lipids (301), carbohydrates (19), cofactors and vitamins (25), nucleotides (27), peptides (20), energy-related metabolites (7), and partially characterized molecules (9).

### 4.3. Statistical Analysis

Descriptive and clinical data analyses were performed using Stata 16 (StataCorp https://www.stata.com/). Continuous variables were summarized as means ± standard deviations (SDs). Categorical variables were reported as frequencies and percentages. Differences were assessed using appropriate statistical tests, including *t*-tests, ANOVA, or chi-square tests, with a significance threshold set at *p* < 0.05. Metabolomics data analysis was conducted using Metaboanalyst 6.0 (https://www.metaboanalyst.ca/, accessed on 5 May 2024). Prior to analysis, metabolite concentrations were log-transformed and auto-scaled to normalize variance. Univariate analysis was performed with False Discovery Rate (FDR) correction to identify significantly altered metabolites between metabolic phenotypes. Group separation and classification were evaluated using orthogonal partial least squares-discriminant analysis (OPLS-DA). Variable importance in projection (VIP) scores was used to identify metabolites that contributed most to class separation. Classifier robustness was estimated using a permutation test (1000 iterations). Pathway enrichment and impact analyses were conducted to determine biologically relevant metabolic pathways. Finally, biomarker analysis, including receiver operating characteristic (ROC) curve analysis and area under the curve (AUC) calculation, was used to assess the discriminatory power of selected metabolites. The cutoff point was selected according to the Youden index (sensitivity + specificity − 1). Statistical significance was set at *p* < 0.05.

### 4.4. Ethical Approval

This protocol has been approved by institutional review boards at the QBB (IRB number: E-2022-QF-QBB-RES-ACC-00096-0198) on 13 June 2022 and the Qatar Biomedical Research Institute (QBRI) (QBRI-IRB-2023-53) on 12 October 2022 and was carried out in accordance with the local legislation, institutional requirements, and the Declaration of Helsinki (Code of Ethics of the World Medical Association) for experiments that involved humans. All study participants signed informed consent.

## Figures and Tables

**Table 1:** General baseline characteristics of participants.

	MHO (1427)	MUHO (472)	*p*-Value *	MHNW (781)	MUHNW (68)	*p*-Value ^#^	*p*-Value ^ᵻ^
Female (%)	930 (65.2%)	278 (58.9%)	0.014	452 (57.9%)	28 (41.2%)	0.008	<0.001
Age (years)	43.0 ± 13.3	46.0 ± 10.9	<0.001	30.9 ± 11.3	42.1 ± 11.4	<0.001	<0.001
BMI (Kg/m^2^)	35.1 ± 4.7	35.0 ± 4.1	0.68	21.9 ± 2.2	23.3 ± 1.8	<0.001	<0.001
FPG (mmol/L)	5.6 ± 1.8	6.5 ± 2.7	<0.001	4.8 ± 0.9	6.2 ± 2.7	<0.001	<0.001
HbA1c (%)	5.8 ± 1.1	6.3 ± 1.5	<0.001	5.2 ± 0.7	6.1 ± 1.6	<0.001	<0.001
T-Cholesterol (mmol/L)	4.6 ± 0.8	5.9 ± 0.7	<0.001	4.3 ± 0.6	6.0 ± 0.7	<0.001	<0.001
HDL (mmol/L)	1.5 ± 0.3	1.1 ± 0.2	<0.001	1.6 ± 0.4	1.1 ± 0.3	<0.001	<0.001
LDL (mmol/L)	2.6 ± 0.7	3.7 ± 0.7	<0.001	2.3 ± 0.5	3.9 ± 0.7	<0.001	<0.001
Triglycerides (mmol/L)	1.1 ± 0.4	2.1 ± 0.7	<0.001	0.8 ± 0.3	2.2 ± 0.8	<0.001	<0.001
Insulin (uUI/mL)	12.5 ± 7.8	16.9 ± 8.7	<0.001	6.9 ± 3.4	13.7 ± 8.1	<0.001	<0.001
CRP (mg/L)	7.0 ± 5.3	8.1 ± 6.1	<0.001	4.3 ± 3.3	5.4 ± 4.1	0.011	<0.001
AST (U/L)	19.2 ± 7.5	20.6 ± 8.8	<0.001	18.8 ± 7.5	20.9 ± 7.3	0.025	<0.001
ALT (U/L)	22.3 ± 14.3	27.6 ± 16.6	<0.001	17.3 ± 11.9	27.4 ± 16.9	<0.001	<0.001
DBP (mmHg)	68.0 ± 10.2	72.3 ± 10.9	<0.001	62.6 ± 8.4	71.3 ± 9.9	<0.001	<0.001
SBP (mmHg)	118.4 ± 14.7	122.7 ± 14.3	<0.001	105.1 ± 12.2	115.6 ± 15.8	<0.001	<0.001
Pulse (BPM)	70.6 ± 9.6	73.8 ± 9.9	<0.001	70.7 ± 10.3	73.2 ± 10.8	0.050	<0.001
Weight (Kg)	92.1 ± 15.1	92.9 ± 14.3	0.32	59.7 ± 9.4	64.3 ± 8.2	<0.001	<0.001
HOMA-IR	3.3 ± 2.9	5.0 ± 3.9	<0.001	1.5 ± 0.9	3.8 ± 3.5	<0.001	<0.001
Fat (%)	44.5 ± 7.0	43.5 ± 7.1	0.008	27.7 ± 8.5	32.6 ± 7.7	<0.001	<0.001
Prediabetes % FPG	206 (14.4%)	119 (25.2%)	<0.001	28 (3.6%)	14 (20.6%)	<0.001	<0.001
Diabetes % FPG	188 (13.2%)	99 (21.0%)	<0.001	17 (2.2%)	10 (14.7%)	<0.001	<0.001
Hypertension %	327 (22.9%)	164 (34.7%)	<0.001	37 (4.7%)	16 (23.5%)	<0.001	<0.001
Insulin Resistance %	867 (60.8%)	457 (96.8%)	<0.001	117 (15.0%)	62 (91.2%)	<0.001	<0.001
Hypertriglyceridemia %	73 (5.1%)	397 (84.1%)	<0.001	9 (1.2%)	55 (80.9%)	<0.001	<0.001

Data is represented as means ± SD or proportions. FPG: fasting plasma glucose; HDL: high-density lipoprotein; LDL: low-density lipoprotein; DBP, diastolic blood pressure; SBP, systolic blood pressure; HOMA-IR: homeostasis model assessment for insulin resistance; HbA1c: glycated hemoglobin A1C; BMI: body mass index, MHO: metabolically healthy obese, MUHO: metabolically unhealthy obese, MHNW: metabolically healthy normal-weight, MUHNW: metabolically unhealthy normal-weight. *p* values marked with * indicate comparison between MHO and MUHO. *p* values marked with ^#^ indicate comparison between MHNW and MUHNW. *p* values marked ^ᵻ^ with indicate comparison across all subgroups.

**Table 2:** (a) Logistic regression analysis between various clinical factors and MUHO in females; (b) Logistic regression analysis between various clinical factors and MUHO in males; (c) Logistic regression analysis between various clinical factors and MUHNW in females; (d) Logistic regression analysis between various clinical factors and MUHNW in males.

(a)		
**Characteristics**	**OR (95% CI)**	***p*** **value**
Cpeptide (ng/mL)	2.02 (1.70–2.42)	<0.001
Potassium (mmol/L)	1.73 (1.09–2.77)	0.02
Albumin (g/L)	1.08 (1.03–1.14)	0.001
Total Protein (g/L)	1.04 (1.002–1.09)	0.038
C reactive protein (mg/L)	1.03 (1.01–1.06)	0.004
Folate (nmol/L)	1.02 (1.006–1.04)	0.009
TIBC (μmol/L)	1.01 (1.003–1.03)	0.015
Ferritin (μg/L)	1.005 (1.002–1.008)	<0.001
Uric Acid (μmol/L)	1.003 (1.001–1.006)	0.008
Estradiol (pmo/L)	0.99 (0.998–0.999)	0.004
Urea (mmol/L)	0.85 (0.74–0.97)	0.024
Free thyroxine (pmol/L)	0.83 (0.77–0.90)	<0.001
(b)		
**Characteristics**	**OR (95% CI)**	***p*** **value**
Cpeptide (ng/mL)	1.66 (1.37–2.01)	<0.001
Albumin (g/L)	1.06 (1.004–1.12)	0.035
Alkaline Phosphatase (U/L)	1.01 (1.001–1.02)	0.028
Uric Acid (μmol/L)	1.003 (1.001–1.006)	0.004
Ferritin (μg/L)	1.003 (1.001–1.005)	<0.001
Estradiol (pmol/L)	0.98 (0.98–0.99)	<0.001
VitD (ng/mL)	0.97 (0.95–0.99)	0.016
Bicarbonate (mmol/L)	0.92 (0.85–0.99)	0.037
Free thyroxine (pmol/L)	0.82 (0.75-.91)	<0.001
(c)		
**Characteristics**	**OR (95% CI)**	***p*** **value**
Cpeptide (ng/mL)	8.57 (3.97–18.50)	<0.001
TSH (mIU/L)	1.26 (1.02–1.55)	0.026
Uric Acid (μmol/L)	1.008 (1.007–1.016)	0.047
Estradiol (pmol/L)	0.99 (0.996–0.999)	0.012
Ftriiodothyronine (pmol/L)	0.33 (0.17–0.64)	0.001
(d)		
**Characteristics**	**OR (95% CI)**	***p*** **value**
Cpeptide (ng/mL)	5.78 (3.05–10.95)	<0.001
Total Protein (g/L)	1.17 (1.04–1.33)	0.01
Uric Acid (μmol/L)	1.009 (1.002–1.01)	0.009
Bilirubin (μmol/L)	0.90 (0.82–0.99)	0.04
Testosterone (nmol/L)	0.89 (0.83–0.96)	0.003
Free thyroxine (pmol/L)	0.62 (0.49–0.78)	<0.001
Urea (mmol/L)	0.41 (0.26–0.65)	<0.001

**Table 3:** Differentially expressed metabolites between MHO and MUHO samples.

Metabolites	FC	FDR
oleoyl-linoleoyl-glycerol (18:1/18:2) [2]	2.33	1.2 × 10^−9^
1-palmitoleoylglycerol (16:1) *	2.3	5.10 × 10^−7^
1-stearoyl-2-oleoyl-GPE (18:0/18:1)	2.15	2.75 × 10^−9^
1-palmitoyl-2-linoleoyl-GPE (16:0/18:2)	2.01	1.66 × 10^−11^
1-stearoyl-2-linoleoyl-GPE (18:0/18:2) *	1.9	8.7 × 10^−10^
1-palmitoyl-GPI (16:0)	1.82	6.45 × 10^−8^
1-palmitoyl-2-palmitoleoyl-GPC (16:0/16:1) *	1.79	8.7 × 10^−10^
1-palmitoyl-2-docosahexaenoyl-GPE (16:0/22:6) *	1.79	2.75 × 10^−9^
1-palmitoyl-2-arachidonoyl-GPI (16:0/20:4) *	1.78	1.13 × 10^−7^
1-linoleoylglycerol (18:2)	1.75	1.45 × 10^−8^
1-palmitoyl-2-arachidonoyl-GPE (16:0/20:4) *	1.69	2.8 × 10^−9^
1-stearoyl-GPE (18:0)	1.68	3.15 × 10^−14^
1-palmitoyl-2-linoleoyl-GPI (16:0/18:2)	1.68	1.14 × 10^−10^
1-linoleoyl-GPE (18:2) *	1.64	1.16 × 10^−11^
1-palmitoyl-GPE (16:0)	1.63	8.03 × 10^−12^
1-stearoyl-2-docosahexaenoyl-GPC (18:0/22:6)	1.57	1.14 × 10^−10^
1-stearoyl-2-oleoyl-GPC (18:0/18:1)	1.57	1.2 × 10^−9^
1-stearoyl-2-arachidonoyl-GPE (18:0/20:4)	1.54	3.56 × 10^−8^
1-palmitoleoyl-GPC (16:1) *	1.5	1.46 × 10^−8^
5alpha-pregnan-3beta,20alpha-diol monosulfate (2)	−1.83	0.007

FC: Fold Change; FDR: False Discovery Rate. * Indicates metabolites with putative identification based on high-confidence spectral matching but lacking confirmation by authentic standards.

**Table 4:** Differentially expressed metabolites between MHO and MHNW samples.

Metabolites	FC	FDR
metabolonic lactone sulfate	2.15	2.01 × 10^−13^
4-hydroxyglutamate	2.09	2.03 × 10^−7^
orotidine	1.94	0.03
N-methylhydroxyproline **	1.698	0.03
1-dihomo-linolenylglycerol (20:3)	1.6474	0.0002
1-palmitoleoylglycerol (16:1) *	1.6184	0.003
1-arachidonylglycerol (20:4)	1.5948	0.001
nisinate (24:6n3)	1.5439	0.002
4-hydroxyphenylacetylglutamine	1.509	0.001
pregnenolone sulfate	−1.59	5 × 10^−6^
glyco-beta-muricholate **	−1.94	0.0007
5alpha-pregnan-3beta,20alpha-diol disulfate	−2.01	2.11 × 10^−5^
pregnanolone/allopregnanolone sulfate	−2.07	0.001
pregnanediol-3-glucuronide	−2.23	2.9 × 10^−6^
5alpha-pregnan-3beta,20beta-diol monosulfate (1)	−2.37	3.44 × 10^−6^
5alpha-pregnan-diol disulfate	−2.53	2.48 × 10^−5^
5alpha-pregnan-3beta,20alpha-diol monosulfate (2)	−2.58	2.14 × 10^−5^

FC: Fold Change; FDR: False Discovery Rate. * Indicates metabolites with putative identification based on high-confidence spectral matching but lacking confirmation by authentic standards. ** Indicates compounds for which authentic standards are unavailable; however, the identities are considered reasonably confident based on the available spectral and annotation data.

## Data Availability

Anthropometric, demographic, and clinical data of the participants were obtained from the Qatar Biobank Cohort upon the submission of a proposal and approval by the IRB. We do not have permission to share the data, and, thus, no permission can be provided. The authors did not receive any special privileges in accessing the data used in the study. Any Researcher can access the data upon the submission of a proposal and approval of the IRB. The data can be accessed by submitting an application through the QBB website (https://www.qphi.org.qa/cohorts-and-disease-based-studies) (accessed on 18 April 2023). Researchers need to create an account to be able to apply.

## Supplementary Materials

The following supporting information can be downloaded at: https://www.mdpi.com/article/10.3390/ijms27104555/s1.
