# Supplementary material for: Metabolomic Pathways Distinguishing Metabolically Healthy and Unhealthy Obesity from Normal-Weight: A Cross-Sectional Study
Source: Int J Mol Sci. 2026 May 19;27(10):4555. doi: 10.3390/ijms27104555 (PMC13207990; doi:10.3390/ijms27104555)
Supplement: Supplementary file 1 [file ijms-27-04555-s001.zip › ijms-4257013-supplementary.pdf]

Metabolomic Pathways Distinguishing Metabolically Healthy and Unhealthy Obesity from Normal Weight: A Cross-Sectional Study

Neyla S. AL Akl [1], Olfa Khalifa [1], Abdelilah Arredouani [1,2]

[1] Diabetes Research Center, Qatar Biomedical Research Institute (QBRI), Hamad Bin Khalifa University (HBKU), Qatar Foundation, Doha, Qatar.
[2] College of Health and Life Sciences, Hamad Bin Khalifa University (HBKU), Qatar Foundation, Doha, Qatar.

Correspondence: aarredouani@hbku.edu.qa

Supplemental Figure S1

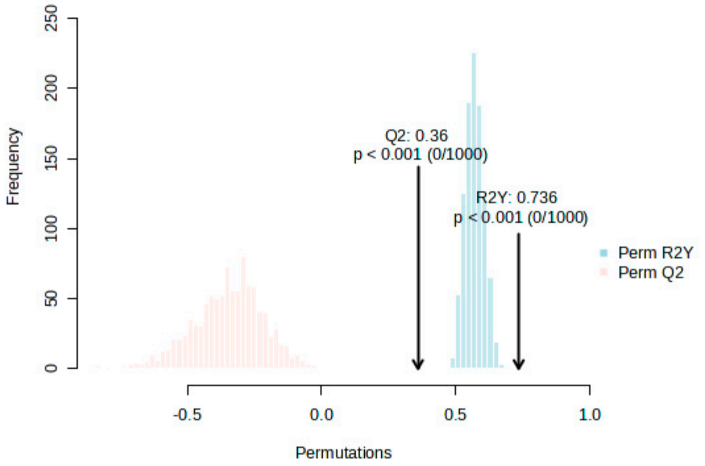

Figure S1: OPLS-DA Model Permutation Test. Permutation test results validating the OPLS-DA model discriminating between metabolically healthy obese (MHO) and metabolically unhealthy obese (MUHO) groups.

Supplemental Figure S2

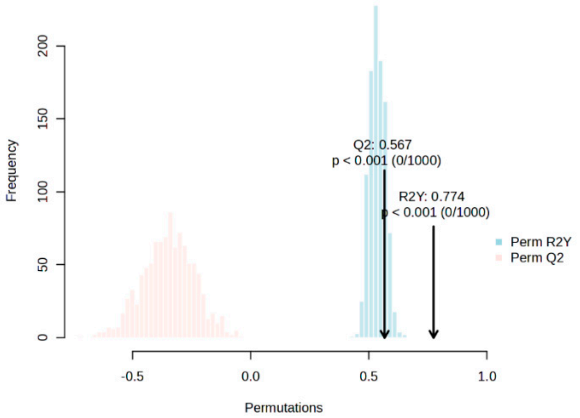

Figure S2: OPLS-DA Model Permutation Test. Permutation test results validating the OPLS-DA model discriminating between metabolically healthy obese (MHO) and metabolically healthy normal weight groups (MHNW).

A

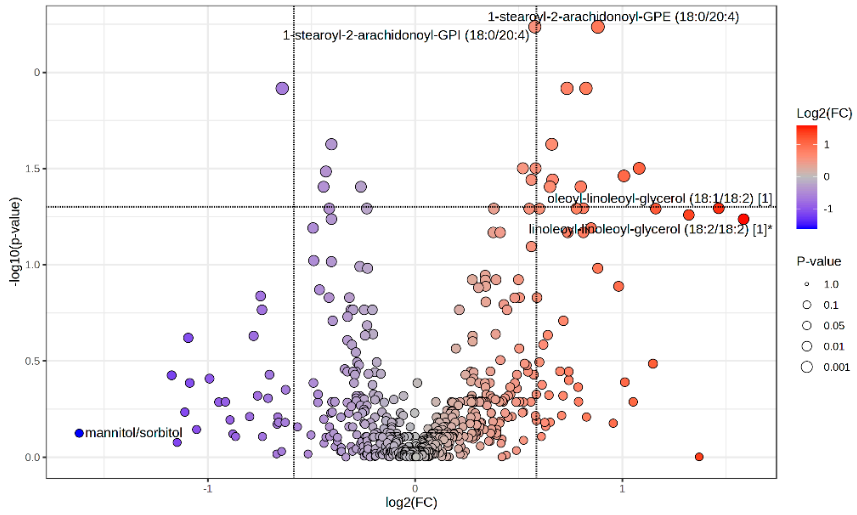

B

Differentially expressed metabolites between MHO and MUHNW samples		
	FC	FDR
1-stearoyl-2-arachidonoyl-GPE (18:0/20:4)	1.84	0.01
1-linoleoyl-GPE (18:2)*	1.77	0.01
1-stearoyl-GPE (18:0)	1.66	0.01
N1-methylinosine	-1.55	0.01
1-stearoyl-2-linoleoyl-GPI (18:0/18:2)	1.58	0.02
1-stearoyl-2-linoleoyl-GPE (18:0/18:2)*	2.12	0.03
1-palmitoyl-2-linoleoyl-GPE (16:0/18:2)	2.01	0.03
1-palmitoyl-2-dihomo-linolenoyl-GPC (16:0/20:3n3 or 6)*	1.58	0.04
1-palmitoyl-2-linoleoyl-GPI (16:0/18:2)	1.74	0.04
1-palmitoyl-GPE (16:0)	1.57	0.04

C

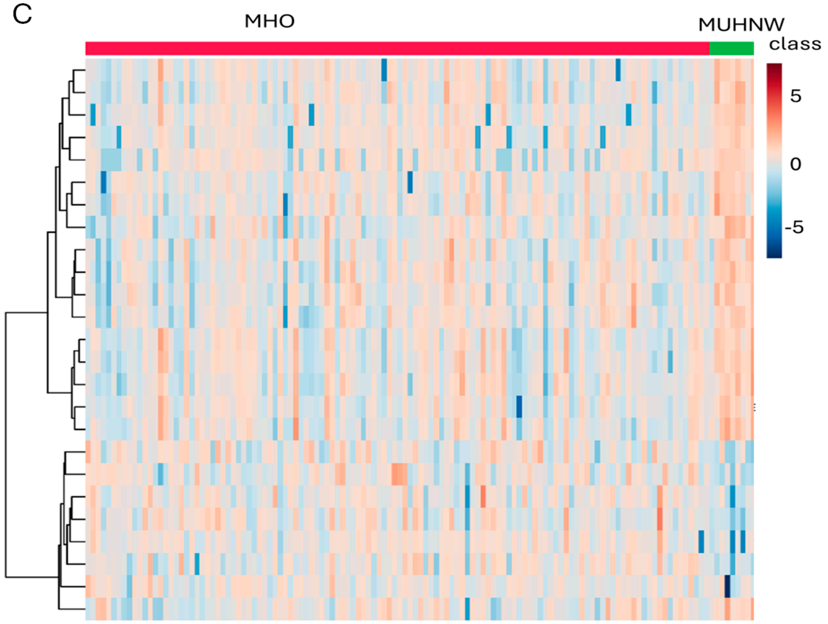

Figure S3. Differential plasma metabolite expression between MHO and MUHNW groups. (A) The volcano plot illustrates the $\log_2$ fold change (*x*-axis) versus the $-\log_{10}$ adjusted *p*-value (*y*-axis) for all detected metabolites. Color represents a continuous gradient of $\log_2$(FC) (red: higher in MUHNW; blue: lower in MUHNW), while point size reflects the magnitude of statistical significance (*p*-value). The horizontal dashed line indicates the significance threshold (FDR = 0.05; $-\log_{10} \approx 1.3$), and vertical dashed lines indicate the fold change threshold (FC = 1.5; $\log_2 FC \approx \pm 0.58$). Metabolites located in the upper right and upper left regions exceed both thresholds, corresponding to significantly upregulated and downregulated metabolites in MUHO, respectively. (B) Differentially expressed metabolites between MHO and MUHNW samples. (C) The heatmap represents the supervised hierarchical clustering of the top 25 differentially expressed metabolites across MHO and MUHNW individuals. Blue shading indicates lower expression, and orange/brown shading indicates higher expression in the MHO group. *Indicates metabolites with putative identification based on high-confidence spectral matching but lacking confirmation by authentic standards.

Supplemental Figure S4

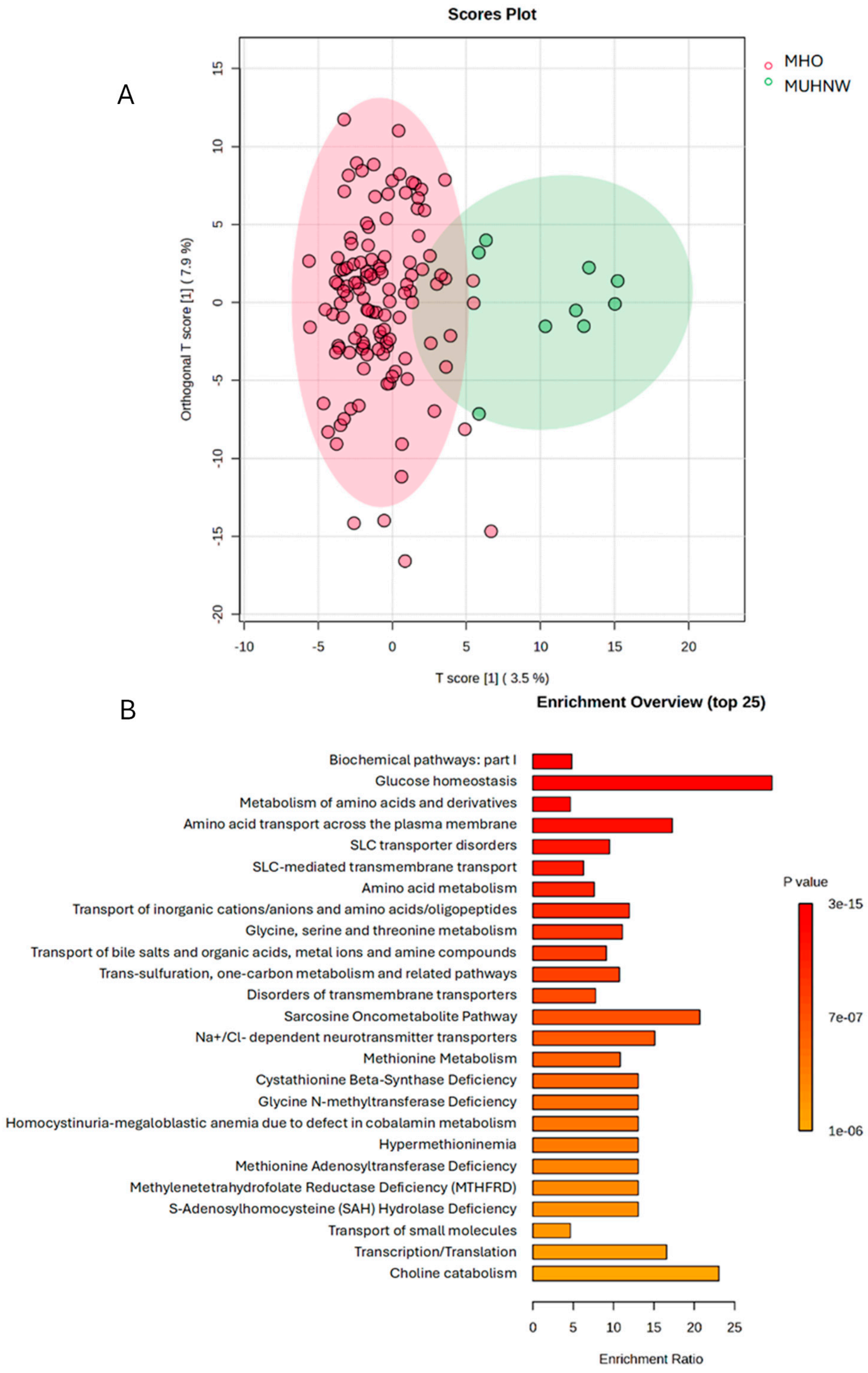

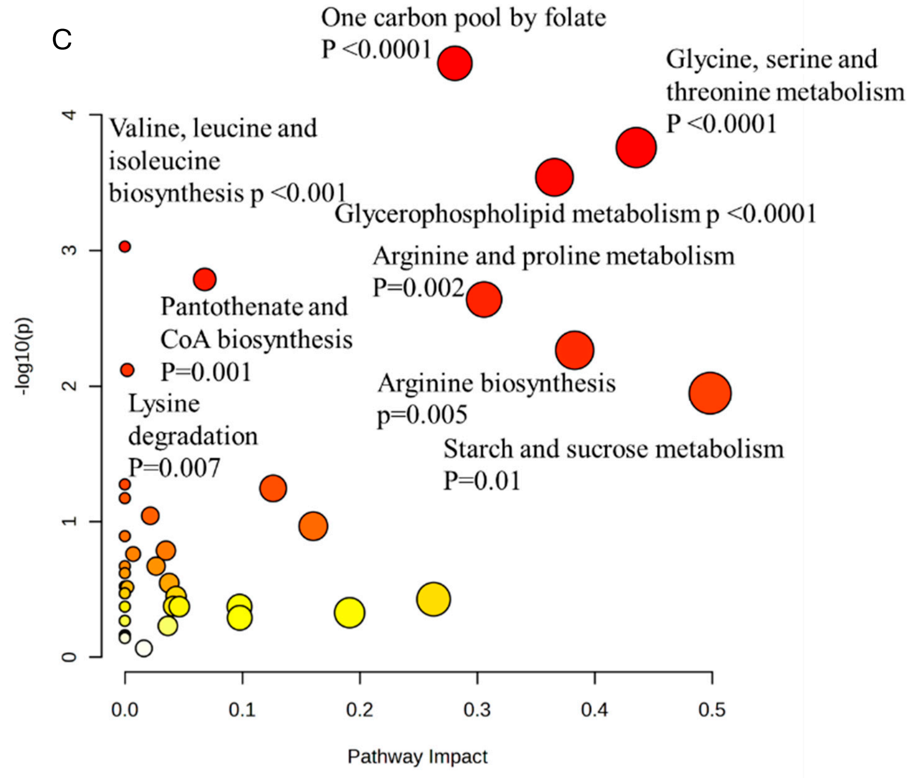

Figure S4. Chemometric analysis and pathway enrichment of metabolomic differences between MHO and MUHNW groups. (A) Orthogonal Partial Least Squares Discriminant Analysis (OPLS-DA) score plot illustrating partial separation between MHO and MUHNW samples. (B) Bar plot representing the top enriched metabolic pathways identified using RaMP-DB, highlighting pathways significantly associated with differential metabolites ($p < 0.05$). (C) Pathway impact analysis based on KEGG and HMDB databases showing the relative influence of key metabolic pathways in distinguishing MHO from MUHNW phenotypes. Pathways with significant p-values ($p < 0.05$) are emphasized.

Supplementary Table S1: Metabolites with VIP >1 based on OrthoPLDA analysis between MHO and MUHO	
Metabolites	VIP
1-stearoyl-GPE (18:0)	3.23
1-palmitoyl-GPE (16:0)	3.04
1-palmitoyl-2-linoleoyl-GPE (16:0/18:2)	3.03
1-linoleoyl-GPE (18:2)*	2.87
1-palmitoyl-2-arachidonoyl-GPE (16:0/20:4)*	2.81
1-palmitoyl-2-docosahexaenoyl-GPE (16:0/22:6)*	2.78
1-stearoyl-2-oleoyl-GPE (18:0/18:1)	2.77
1-stearoyl-2-linoleoyl-GPE (18:0/18:2)*	2.72
1-palmitoyl-2-palmitoleoyl-GPC (16:0/16:1)*	2.68
1-stearoyl-2-docosahexaenoyl-GPC (18:0/22:6)	2.62
1-arachidonoyl-GPE (20:4n6)*	2.60
1-palmitoyl-2-oleoyl-GPE (16:0/18:1)	2.59
oleoyl-linoleoyl-glycerol (18:1/18:2) [2]	2.55
1-stearoyl-2-arachidonoyl-GPE (18:0/20:4)	2.54
1-stearoyl-2-oleoyl-GPC (18:0/18:1)	2.54
1-palmitoyl-2-linoleoyl-GPI (16:0/18:2)	2.50
1-carboxyethylphenylalanine	2.46
1-palmitoyl-2-oleoyl-GPC (16:0/18:1)	2.44
pyruvate	2.41
1-palmitoyl-2-docosahexaenoyl-GPC (16:0/22:6)	2.32
1-palmitoyl-GPC (16:0)	2.30
1-palmitoleoyl-GPC (16:1)*	2.27
1-palmitoyl-2-arachidonoyl-GPI (16:0/20:4)*	2.25
serine	2.23
1-stearoyl-GPC (18:0)	2.22
1-linoleoylglycerol (18:2)	2.21
1,2-dipalmitoyl-GPC (16:0/16:0)	2.20
valine	2.18
glycerophosphoethanolamine	2.15
1-palmitoleoylglycerol (16:1)*	2.10
1-stearoyl-2-arachidonoyl-GPI (18:0/20:4)	2.08
1-stearoyl-2-linoleoyl-GPI (18:0/18:2)	2.08
oleoyl-linoleoyl-glycerol (18:1/18:2) [1]	2.07
1-linolenoyl-GPC (18:3)*	2.07
1-myristoyl-2-palmitoyl-GPC (14:0/16:0)	1.99
2-aminoadipate	1.94
alpha-hydroxyisovalerate	1.92
N-palmitoyl-sphingosine (d18:1/16:0)	1.91
1-myristoyl-2-arachidonoyl-GPC (14:0/20:4)*	1.91

3-(4-hydroxyphenyl)lactate	1.89
sphingomyelin (d18:0/20:0, d16:0/22:0)*	1.89
linoleoyl-linoleoyl-glycerol (18:2/18:2) [1]*	1.88
1-palmitoyl-GPI (16:0)	1.88
sphingomyelin (d18:0/18:0, d19:0/17:0)*	1.87
1-palmitoyl-2-arachidonoyl-GPC (16:0/20:4n6)	1.84
metabolonic lactone sulfate	1.83
1-stearoyl-2-arachidonoyl-GPC (18:0/20:4)	1.82
2-hydroxy-3-methylvalerate	1.80
5-methylthioadenosine (MTA)	1.80
palmitoylcarnitine (C16)	1.79
1-oleoyl-GPC (18:1)	1.78
N-stearoyl-sphingosine (d18:1/18:0)*	1.78
stearoylcarnitine (C18)	1.76
3-methyl-2-oxobutyrate	1.74
1-palmitoyl-2-linoleoyl-GPC (16:0/18:2)	1.74
1-stearoyl-2-linoleoyl-GPC (18:0/18:2)*	1.73
3-methyl-2-oxovalerate	1.73
1-palmitoyl-2-oleoyl-GPI (16:0/18:1)*	1.72
N-acetylleucine	1.68
malate	1.66
gamma-glutamylvaline	1.65
cysteine	1.63
gamma-glutamylglutamine	1.62
cerotoylcarnitine (C26)*	1.62
retinol (Vitamin A)	1.62
gamma-glutamylisoleucine*	1.61
1-linoleoyl-GPC (18:2)	1.60
2,3-dihydroxy-5-methylthio-4-pentenoate (DMTPA)*	1.60
gamma-tocopherol/beta-tocopherol	1.59
alpha-hydroxyisocaproate	1.59
N-acetyltryptophan	1.58
alpha-tocopherol	1.58
1-linoleoyl-GPI (18:2)*	1.58
4-methyl-2-oxopentanoate	1.57
1-oleoyl-GPE (18:1)	1.55
proline	1.54
5alpha-pregnan-3beta,20alpha-diol monosulfate (2)	1.54
1-linolenoylglycerol (18:3)	1.53
3-hydroxy-2-ethylpropionate	1.53
urate	1.53

1-linoleoyl-2-arachidonoyl-GPC (18:2/20:4n6)*	1.52
aconitate [cis or trans]	1.49
behenoyl sphingomyelin (d18:1/22:0)*	1.48
1-oleoyl-2-docosahexaenoyl-GPC (18:1/22:6)*	1.48
16-hydroxypalmitate	1.47
indolelactate	1.46
2-hydroxy-4-(methylthio)butanoic acid	1.46
1-arachidonoyl-GPC (20:4n6)*	1.45
N-acetylputrescine	1.41
cysteine-glutathione disulfide	1.41
4-hydroxyglutamate	1.41
alpha-hydroxycaproate	1.39
7-alpha-hydroxy-3-oxo-4-cholestenoate (7-Hoca)	1.39
1-(1-enyl-palmitoyl)-2-oleoyl-GPC (P-16:0/18:1)*	1.36
pantothenate	1.36
1-palmitoyl-2-dihomo-linolenoyl-GPC (16:0/20:3n3 or 6)*	1.36
N-acetylglycine	1.35
1-arachidonoyl-GPI (20:4)*	1.33
gamma-glutamylcitrulline*	1.32
N-acetylisoleucine	1.32
androstenediol (3beta,17beta) disulfate (1)	1.30
sphingomyelin (d18:2/24:2)*	1.29
sphingomyelin (d18:1/20:0, d16:1/22:0)*	1.27
gamma-glutamylleucine	1.27
ribitol	1.26
phenyllactate (PLA)	1.25
pregnanediol-3-glucuronide	1.25
beta-hydroxyisovalerate	1.25
2-hydroxybutyrate/2-hydroxyisobutyrate	1.24
5-hydroxylysine	1.24
pipecolate	1.23
N-acetylphenylalanine	1.23
1-dihomo-linolenylglycerol (20:3)	1.22
phenylacetate	1.21
glycerophosphorylcholine (GPC)	1.20
1-palmitoylglycerol (16:0)	1.19
gamma-glutamylthreonine	1.18
gamma-glutamylglycine	1.18
1-arachidonylglycerol (20:4)	1.17
phenylacetylglutamine	1.17
beta-citrylglutamate	1.16

picolinoylglycine	1.16
N2,N2-dimethylguanosine	1.16
palmitoylcholine	1.16
N-acetylvaline	1.15
glucose	1.13
N6-carbamoylthreonyladenosine	1.13
5alpha-androstan-3beta,17beta-diol disulfate	1.12
cysteinylglycine	1.12
N1-methyladenosine	1.11
sphingomyelin (d18:1/14:0, d16:1/16:0)*	1.10
1,2-dilinoleoyl-GPC (18:2/18:2)	1.08
p-cresol glucuronide*	1.08
ethylmalonate	1.08
propionylcarnitine (C3)	1.06
1-stearoyl-GPI (18:0)	1.06
xanthine	1.06
sphingomyelin (d18:1/21:0, d17:1/22:0, d16:1/23:0)*	1.05
N6-methyladenosine	1.04
* Indicates metabolites with putative identification based on high-confidence spectral matching but lacking confirmation by authentic standards. ** Indicates compounds for which authentic standards are unavailable; however, the identities are considered reasonably confident based on the available spectral and annotation data.	

Supplementary Table S2: Metabolites with VIP >1 based on OrthoPLDA analysis between MHNW and MHO	
Metabolites	VIP
metabolonic lactone sulfate	3.06
sphingomyelin (d18:0/18:0, d19:0/17:0)*	2.75
hydroxyasparagine**	2.73
gamma-glutamyltyrosine	2.57
4-hydroxyglutamate	2.44
dihomo-linolenate (20:3n3 or n6)	2.42
N-acetylglycine	2.42
sphingomyelin (d18:2/14:0, d18:1/14:1)*	2.30
1-(1-enyl-stearoyl)-2-linoleoyl-GPE (P-18:0/18:2)*	2.28
1-(1-enyl-palmitoyl)-2-linoleoyl-GPC (P-16:0/18:2)*	2.27
sphingomyelin (d18:0/20:0, d16:0/22:0)*	2.21
xanthine	2.16
5alpha-pregnan-3beta,20beta-diol monosulfate (1)	2.16
gamma-glutamylvaline	2.15
mannose	2.14
5-hydroxylysine	2.13
1-(1-enyl-palmitoyl)-2-oleoyl-GPC (P-16:0/18:1)*	2.13
pregnanediol-3-glucuronide	2.12
pyruvate	2.11
2,3-dihydroxy-5-methylthio-4-pentenoate (DMTPA)*	2.09
pregnenediol sulfate (C21H34O5S)*	2.07
pregnenolone sulfate	2.06
gamma-glutamylglutamate	2.06
eicosanedioate (C20-DC)	2.04
5alpha-pregnan-3beta,20alpha-diol monosulfate (2)	2.03
androsterone sulfate	2.02
5alpha-pregnan-diol disulfate	2.02
5alpha-pregnan-3beta,20alpha-diol disulfate	2.01
1-stearoyl-2-arachidonoyl-GPC (18:0/20:4)	1.97
glucose	1.94
1-myristoyl-2-arachidonoyl-GPC (14:0/20:4)*	1.93
cortolone glucuronide (1)	1.92
gamma-glutamylphenylalanine	1.92
sphingomyelin (d18:2/21:0, d16:2/23:0)*	1.90
serine	1.87
nisinate (24:6n3)	1.86
1-dihomo-linolenylglycerol (20:3)	1.83
5-methylthioadenosine (MTA)	1.82
1-palmitoyl-2-dihomo-linolenoyl-GPC (16:0/20:3n3 or 6)*	1.82

N6-carbamoylthreonyladenosine	1.81
hypoxanthine	1.81
1-linoleoyl-GPE (18:2)*	1.80
picolinoylglycine	1.78
dehydroepiandrosterone sulfate (DHEA-S)	1.77
7-alpha-hydroxy-3-oxo-4-cholestenoate (7-Hoca)	1.76
1-palmitoyl-2-palmitoleoyl-GPC (16:0/16:1)*	1.76
epiandrosterone sulfate	1.74
1-linoleoyl-GPC (18:2)	1.72
3-(4-hydroxyphenyl)lactate	1.72
linoleoylcholine*	1.71
quinolinate	1.70
5,6-dihydrouracil	1.69
1-palmitoyl-2-arachidonoyl-GPC (16:0/20:4n6)	1.69
1-stearoyl-2-docosahexaenoyl-GPC (18:0/22:6)	1.69
pregnanolone/allopregnanolone sulfate	1.68
fructose	1.67
2-aminoadipate	1.66
2R,3R-dihydroxybutyrate	1.66
5alpha-androstan-3alpha,17alpha-diol monosulfate	1.66
gamma-glutamylisoleucine*	1.65
fructosyllysine	1.64
1-carboxyethylphenylalanine	1.64
glyco-beta-muricholate**	1.64
1-(1-enyl-palmitoyl)-2-linoleoyl-GPE (P-16:0/18:2)*	1.64
carnitine	1.63
sphingomyelin (d18:1/18:1, d18:2/18:0)	1.63
androstenediol (3alpha, 17alpha) monosulfate (3)	1.62
arabitol/xylitol	1.61
1-arachidonylglycerol (20:4)	1.60
N2,N2-dimethylguanosine	1.59
eicosenedioate (C20:1-DC)*	1.57
valine	1.56
sphingomyelin (d18:2/23:1)*	1.56
kynurenine	1.55
C-glycosyltryptophan	1.54
urate	1.53
1-palmitoleoylglycerol (16:1)*	1.53
2-O-methylascorbic acid	1.53
1-linoleoyl-2-linolenoyl-GPC (18:2/18:3)*	1.53
N-acetylalanine	1.50

10-heptadecenoate (17:1n7)	1.48
octadecenedioate (C18:1-DC)	1.48
gamma-glutamylleucine	1.48
4-hydroxyphenylacetylglutamine	1.48
glycerol	1.47
pregnenediol disulfate (C21H34O8S2)*	1.47
caprate (10:0)	1.46
cysteinylglycine disulfide*	1.46
gamma-glutamyl-alpha-lysine	1.46
dodecanedioate (C12-DC)	1.44
argininate*	1.43
laurylcarnitine (C12)	1.42
1-ribosyl-imidazoleacetate*	1.42
sphingomyelin (d18:1/22:2, d18:2/22:1, d16:1/24:2)*	1.41
21-hydroxypregnenolone disulfate	1.39
sphingomyelin (d17:2/16:0, d18:2/15:0)*	1.37
1,2-dilinoleoyl-GPC (18:2/18:2)	1.37
2-hydroxydecanoate	1.36
sphingomyelin (d18:2/16:0, d18:1/16:1)*	1.35
1-(1-enyl-stearoyl)-2-oleoyl-GPE (P-18:0/18:1)	1.35
palmitoyl ethanolamide	1.35
pregnenetriol sulfate*	1.34
glucuronide of C10H18O2 (7)*	1.34
lysine	1.34
17alpha-hydroxypregnenolone 3-sulfate	1.33
caprylate (8:0)	1.31
3beta,7alpha-dihydroxy-5-cholestenoate	1.31
gamma-glutamyltryptophan	1.30
decanoylcarnitine (C10)	1.30
lactosyl-N-palmitoyl-sphingosine (d18:1/16:0)	1.30
1-(1-enyl-oleoyl)-GPE (P-18:1)*	1.29
erythronate*	1.29
1-(1-enyl-palmitoyl)-GPC (P-16:0)*	1.29
1-(1-enyl-stearoyl)-GPE (P-18:0)*	1.29
pro-hydroxy-pro	1.27
1-linoleoylglycerol (18:2)	1.27
N-acetylproline	1.26
kynurenate	1.26
1-carboxyethylvaline	1.25
gamma-glutamylglutamine	1.25
palmitoleate (16:1n7)	1.25

aconitate [cis or trans]	1.25
docosapentaenoate (n6 DPA; 22:5n6)	1.24
methylsuccinoylcarnitine	1.24
trigonelline (N'-methylnicotinate)	1.24
N1-methylinosine	1.24
androstenediol (3alpha, 17alpha) monosulfate (2)	1.24
cysteine-glutathione disulfide	1.23
1-linoleoyl-GPA (18:2)*	1.23
dihomo-linoleate (20:2n6)	1.23
myristoyl dihydrosphingomyelin (d18:0/14:0)*	1.23
stearoyl sphingomyelin (d18:1/18:0)	1.23
2-aminooctanoate	1.21
oleoyl-linoleoyl-glycerol (18:1/18:2) [1]	1.21
oleoyl-linoleoyl-glycerol (18:1/18:2) [2]	1.21
tridecenedioate (C13:1-DC)*	1.20
1-arachidonoyl-GPC (20:4n6)*	1.20
3b-hydroxy-5-cholenoic acid	1.19
adrenate (22:4n6)	1.18
sphingomyelin (d18:1/19:0, d19:1/18:0)*	1.18
N1-Methyl-2-pyridone-5-carboxamide	1.18
eicosapentaenoate (EPA; 20:5n3)	1.18
branched-chain, straight-chain, or cyclopropyl 10:1 fatty acid (1)*	1.17
oleoyl ethanolamide	1.17
1-arachidonoyl-GPI (20:4)*	1.17
3beta-hydroxy-5-cholestenoate	1.17
biliverdin	1.16
11beta-hydroxyandrosterone glucuronide	1.16
2-hydroxy-4-(methylthio)butanoic acid	1.16
gamma-glutamylcitrulline*	1.16
indolelactate	1.14
tetradecanedioate (C14-DC)	1.14
sphingomyelin (d18:1/14:0, d16:1/16:0)*	1.14
taurodeoxycholic acid 3-sulfate	1.14
hexadecadienoate (16:2n6)	1.13
1-stearoyl-2-arachidonoyl-GPE (18:0/20:4)	1.13
1-(1-enyl-palmitoyl)-2-oleoyl-GPE (P-16:0/18:1)*	1.13
1-oleoyl-GPC (18:1)	1.13
N-acetylleucine	1.12
5-methyluridine (ribothymidine)	1.12
pseudouridine	1.12
hexadecenedioate (C16:1-DC)*	1.12

octadecadienedioate (C18:2-DC)*	1.10
tricosanoyl sphingomyelin (d18:1/23:0)*	1.09
octanoylcarnitine (C8)	1.09
sphingomyelin (d18:1/21:0, d17:1/22:0, d16:1/23:0)*	1.09
phosphate	1.09
stearoylcarnitine (C18)	1.08
1-palmitoyl-2-arachidonoyl-GPI (16:0/20:4)*	1.08
sphingomyelin (d18:2/24:2)*	1.07
oleoylcholine	1.07
1-(1-enyl-palmitoyl)-2-palmitoyl-GPC (P-16:0/16:0)*	1.07
homoarginine	1.07
S-adenosylhomocysteine (SAH)	1.07
N-acetylvaline	1.06
taurolithocholate 3-sulfate	1.05
creatine	1.05
alpha-hydroxyisovalerate	1.05
3-methylhistidine	1.04
docosahexaenoate (DHA; 22:6n3)	1.04
1-palmitoyl-2-linoleoyl-GPC (16:0/18:2)	1.02
3-methylglutarylcarnitine (2)	1.01
cysteine s-sulfate	1.01
1-palmitoleoyl-GPC (16:1)*	1.01
* Indicates metabolites with putative identification based on high-confidence spectral matching but lacking confirmation by authentic standards. ** Indicates compounds for which authentic standards are unavailable; however, the identities are considered reasonably confident based on the available spectral and annotation data.	
